# FOXP3 and miR-155 cooperate to control the invasive potential of human breast cancer cells by down regulating ZEB2 independently of ZEB1

**DOI:** 10.18632/oncotarget.25523

**Published:** 2018-06-12

**Authors:** Cheryl Y. Brown, Sonia Dayan, Soon Wei Wong, Adrian Kaczmarek, Christopher M. Hope, Stephen M. Pederson, Victoria Arnet, Gregory J. Goodall, Darryl Russell, Timothy J. Sadlon, Simon C. Barry

**Affiliations:** ^1^ Discipline of Paediatrics, School of Medicine, Women’s and Children’s Hospital, University of Adelaide, Adelaide, 5006 SA, Australia; ^2^ Molecular Immunology, Robinson Research Institute, School of Medicine, University of Adelaide, Adelaide, 5005 SA, Australia; ^3^ Department of Gastroenterology, WCHN, Adelaide, 5006 SA, Australia; ^4^ Research Centre for Reproductive Health, Robinson Research Institute, School of Medicine, University of Adelaide, Adelaide, 5005 SA, Australia; ^5^ Gene Regulation Laboratory, Centre for Cancer Biology, University of South Australia, Adelaide, 5006 SA, Australia

**Keywords:** FOXP3, miR155, ZEB2, tumour suppressor, EMT

## Abstract

Control of oncogenes, including ZEB1 and ZEB2, is a major checkpoint for preventing cancer, and loss of this control contributes to many cancers, including breast cancer. Thus tumour suppressors, such as FOXP3, which is mutated or lost in many cancer tissues, play an important role in maintaining normal tissue homeostasis. Here we show for the first time that ZEB2 is selectively down regulated by FOXP3 and also by the FOXP3 induced microRNA, miR-155. Interestingly, neither FOXP3 nor miR-155 directly altered the expression of ZEB1. In breast cancer cells repression of ZEB2, independently of ZEB1, resulted in reduced expression of a mesenchymal marker, Vimentin and reduced invasion. However, there was no de-repression of E-cadherin and migration was enhanced. Small interfering RNAs targeting ZEB2 suggest that this was a direct effect of ZEB2 and not FOXP3/miR-155. In normal human mammary epithelial cells, depletion of endogenous FOXP3 resulted in de-repression of ZEB2, accompanied by upregulated expression of vimentin, increased E-cadherin expression and cell morphological changes. We suggest that FOXP3 may help maintain normal breast epithelial characteristics through regulation of ZEB2, and loss of FOXP3 in breast cancer cells results in deregulation of ZEB2.

## INTRODUCTION

A major cause of mortality in breast cancer patients is cancer metastasis [1], a key component of which is activation of epithelial-to mesenchymal transition (EMT) [2], in which cells lose epithelial features and acquire mesenchymal characteristics. However, EMT does have a vital role in normal biological processes including differentiation and development [3], but in cancer it is hijacked and this licenses the cancer cells to disseminate to distant organs. Two proteins with well-established roles in regulating EMT are the transcription factors ZEB1 and ZEB2 [1, 4], high levels of which are associated with enhanced cell motility and invasion through the repression of genes including E-cadherin, which is required to maintain cell-to cell contacts [4], and the induction of genes associated with mesenchymal characteristics including Vimentin [5], an intermediate filament protein, which is required for the formation and function of invadopodia [6, 7] and is crucial for invasion.

ZEB1 and ZEB2 have overlapping roles in cancer metastasis [1, 8, 9] but there is evidence that they also have separate, non-redundant roles in development and cancer [10–14]. While some of the molecular mechanisms that control levels of both of the ZEB proteins have been documented [15–18], there are now new tumour suppressor candidates implicated in the control of ZEB1 and ZEB2. Identifying these regulatory mechanisms will therefore provide important new insights into how these proteins become over-expressed in cancer progression. The transcription factor FOXP3 is widely known to have a crucial role in the development and function of regulatory T cells, helping to maintain immune homeostasis [19–22]. More recently FOXP3 has emerged as a tumour suppressor in breast [23–29] and prostate [28, 30] epithelia, repressing a number of oncogenes including c-myc [30], Ezh2 [25], HER-2/ErbB2 [23] SKP2 [24] and SATB1 [26], while up regulating expression of tumour suppressors *p*21 [27] and LATS2 [28].

Our genome-wide chromatin immunoprecipitation (Chip-on-chip) studies in human Treg cells [31] and other studies in breast cancer cells [32] identified a large number of potential FOXP3 target genes including loci encoding microRNAs (miRs) [31]. MicroRNAs (miRs) are key players in the control of multiple biological processes [33, 34], down regulating genes through mRNA degradation or translational arrest. Previously, we have demonstrated that FOXP3 can function together with the FOXP3-regulated microRNAs, miR-7 and miR-155 to form feed forward loops to tightly control SATB1 levels in breast epithelial cells [26] and in Treg cells [35]. This has raised the possibility that other target genes are finely regulated in this way. In this study we identified ZEB2 as also being regulated by such a feed forward loop involving FOXP3 and the FOXP3-induced microRNA, miR-155. This links FOXP3 tumour suppressor activity in breast epithelia to the regulation of EMT.

As well as having an important role in the normal immune compartment [36–39], miR-155 has been linked to the development and poor prognosis of both lymphoid malignancies [40] and a range of solid tumours of the breast [41, 42] pancreas [43] kidney [44] and lung [45]. Other studies have indicated that miR-155 can also have a protective role in cancer [46–48]. Our finding that miR-155 participates in the control of ZEB2, a key component of the regulatory circuit governing EMT, supports a complex role for miR-155 in cancer, consistent with the notion that cancer progression may be dependent on the interaction of a number of different factors, including the presence of other mutations in the cell, and the loss of master regulators such as FOXP3.

Here we show by overexpression of FOXP3 and/ or miR-155 that in aggressive breast cancer lines ZEB2 is regulated independently of ZEB1, both transcriptionally, by FOXP3 and post-transcriptionally by miR-155. The loss of ZEB2, independently of ZEB1, was accompanied by reduced expression of the mesenchymal marker Vimentin but, surprisingly, not the de-repression of E-cadherin. These findings were reciprocated in normal human mammary epithelial cells, whereby depletion of FOXP3 by lentiviral knockdown, resulted in de-repression of endogenous ZEB2, accompanied by enhanced Vimentin expression and, again rather surprisingly, enhanced expression of E-cadherin. This independent regulation of ZEB2 reveals that the role of ZEB2 may be more complex than has previously been described. Our results suggest that FOXP3 (and FOXP3 regulated miR-155) may help maintain normal breast epithelial characteristics by the specific regulation of ZEB2.

## RESULTS

### FOXP3 directly down regulates ZEB2

**Figure 1:**
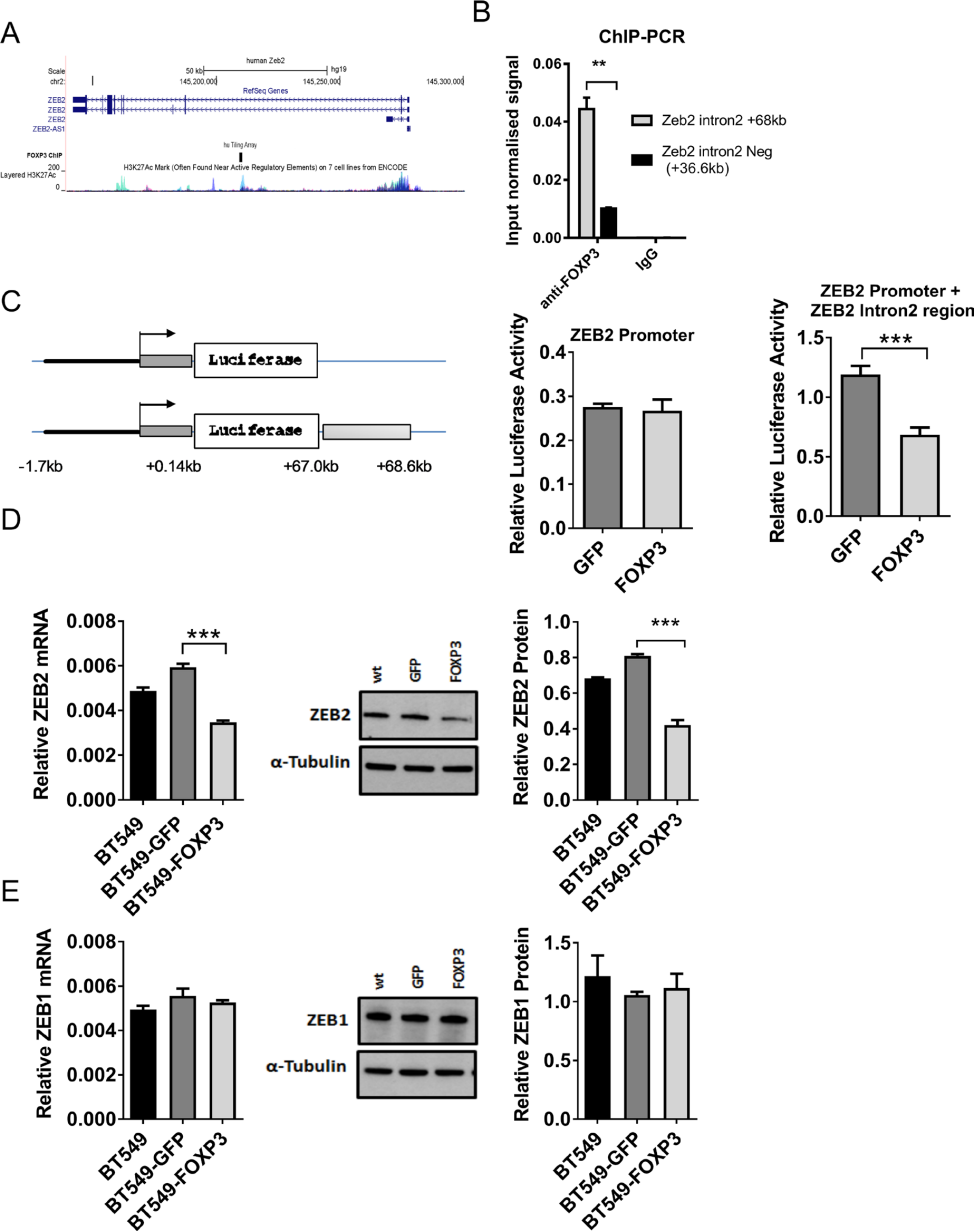
FOXP3 targeting of ZEB2 but not ZEB1 (**A**) Schematic of the human ZEB2 gene (top), FOXP3 binding region (middle) and human ENCODE data for H3K27ac marks (bottom). (**B**) ChIP-PCR on cross-linked material from FOXP3 overexpressing BT549 cells using either rabbit anti-FOXP3 polyclonal Ab or chip grade control rabbit IgG sera. The relative enrichment of target regions in FOXP3-and control IgG immunoprecipitated material relative to input chromatin was calculated using the 2^−ΔΔCT^ method. This input normalised signal was then plotted. A representative experiment, with triplicate samples, of three experiments is shown, mean + SD. A one sample two- tailed *t* test was applied (^**^P < 0.01). (**C**) Schematic representation of the luciferase reporter constructs. Constructs in pGL4.10 incorporating ZEB2 promoter sequences alone (11.7 kb to + 0.1 Kb relative to TS), (Promoter) or the ZEB2 promoter and the FOXP3 binding region in intron 2 (+ 67 kb to + 68.6Kb downstream of the ZEB2 TSS), (Promoter + Intron). The mean normalised luciferase activity from constructs transfected into FOXP3 or GFP overexpressing BT549 cells is shown + SD. *n* = 3. Two tailed Student’s *t* test, ^***^P < 0.001. (**D**) ZEB2 expression in WT, and GFP or FOXP3 overexpressing BT549 cells. Relative abundance of ZEB2 mRNA normalised to reference gene RPL13a is plotted (left). Reactions for quantitative real -time PCR were run in triplicate and the means of the threshold cycles (Cts) were used for quantitation. A standard curve to determine amplification efficiency was generated for ZEB2 and for the reference gene RPL13a mRNAs (see Methods section). The standard curve method for relative quantitation was used to determine the relative abundance of ZEB2 mRNA normalised to the RPL13a reference gene mean + SD (left) Student’s *t* test, ^***^P < 0.001 *n =* 3 experiments. ZEB2 protein by Western blot (middle, shown is a representative experiment) and quantitated relative to α Tubulin (right) mean + SD Student’s *t* test ^***^P < 0.001. *n =* 3 experiments. (**E**) ZEB1 expression in WT, and GFP or FOXP3 overexpressing BT549 cells. Relative abundance of ZEB1 mRNA was quantitated as in (D) above by qRT-PCR using the standard curve method for relative quantitation, and expressed relative to reference gene RPL13A mean + SD (left). ZEB1 protein by western blot (middle, shown is a representative experiment) and quantitated relative to α Tubulin (right) mean + SD. *n =* 3 experiments.

FOXP3 ChIP-on-chip studies performed in our lab [31] identified a region 68 kb downstream of the ZEB2 transcriptional start site (TSS) in Intron 2 of the ZEB2 gene that was significantly bound by FOXP3 (Figure 1A) indicating that ZEB2 was potentially directly regulated by FOXP3. We then confirmed FOXP3 binding to this region of ZEB2 Intron 2 using chip PCR (4 fold enrichment vs. control, Figure 1B). To test whether FOXP3 binding to this region had functional consequences, we assayed the FOXP3-bound region of ZEB2 intron 2 using transcriptional reporter vectors (pGL4.10), containing either the ZEB2 promoter alone or the ZEB2 promoter plus the intron 2 region (Figure 1C). Co- expression of either FOXP3 or GFP with the reporter containing ZEB2 promoter alone in HEK293 cells resulted in no significant difference in luminescence, indicating no effect of FOXP3 on the promoter. However, FOXP3 significantly repressed (40% reduction) luciferase activity expressed from the reporter vector that included the FOXP3 binding site in Intron 2 (Figure 1C), suggesting that FOXP3 binding to target sequences in Intron 2 of the ZEB2 gene reduced its transcription.

To verify that FOXP3 regulates the endogenous ZEB2 gene, we examined the effect of enforced FOXP3 expression in BT549 breast cancer cells, which normally have low levels of FOXP3 [23] and express ZEB2 [49]. Expression of ZEB2 was significantly reduced (mRNA by 41.5% and protein by 48.0%) (Figure 1D) in FOXP3 + BT549 cells, compared with GFP + BT549 cells, indicating that the endogenous ZEB2 gene is regulated by FOXP3 in breast cancer cells. In contrast, FOXP3 had no effect on expression of ZEB1 (Figure 1E). This result suggests that FOXP3 specifically reduces expression of ZEB2 but not ZEB1 and has important implications for the functional contribution of each ZEB protein to the development of breast cancer.

### miR-155 directly down regulates ZEB2 via sites in its 3′UTR

**Figure 2:**
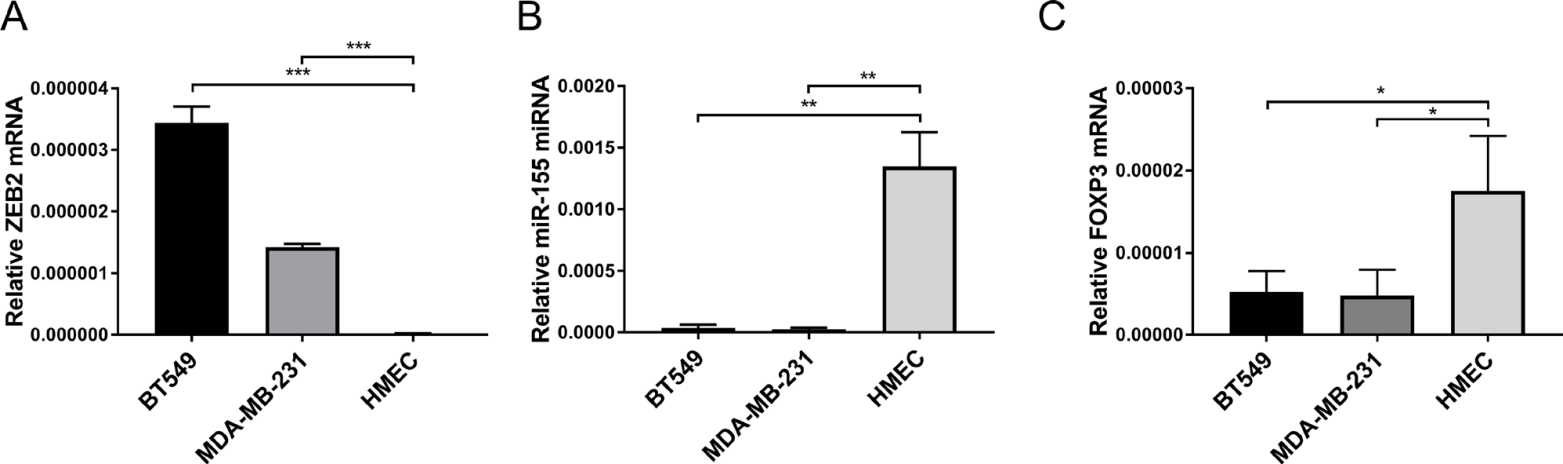
Characterisation of FOXP3, miR-155 and ZEB2 expression patterns in breast cancer and normal breast epithelial cells (**A**) Relative abundance of ZEB2 mRNA normalised to reference gene RPL13A is plotted mean + SEM, unpaired Student’s *t* test, ^***^P < 0.001. Quantitative real -time PCR reactions were in triplicate and the means of the threshold cycles (Cts) were used for quantitation. A standard curve to determine amplification efficiency was generated (see Methods section) for ZEB2 and for the reference gene RPL13a mRNAs. The standard curve method for relative quantitation was used to determine the relative abundance of ZEB2 mRNA normalised to the RPL13a reference gene. 3 frozen stocks for each of BT549, MDA-MB-231 and HMECS cells were cultured as described in Methods section and RNA was isolated. (**B**) Relative abundance of miR-155 miRNA normalised to reference RNU-24 is plotted mean + SEM, unpaired Student’s *t* test, ^**^P < 0.01. Reactions for quantitative real -time PCR were run in triplicate, standard curves to determine amplification efficiency were determined for miR-155 and RNU-24 reference and the standard curve method for determination of relative abundance of mRNA was as in (A) above. 3 frozen stocks for each of BT549, MDA-MB-231 and HMECS were as in (A) above. (**C**) Relative abundance of FOXP3 mRNA normalised to reference gene RPL13a is plotted mean + SEM, unpaired Student’s *t* test, ^*^P < 0.05. Reactions for quantitative real -time PCR were run in triplicate, standard curves to determine amplification efficiency were determined for FOXP3 and RPL13a reference and the standard curve method for determination of relative abundance of mRNA was as in (A) above. 3 frozen stocks for each of BT549, MDA-MB-231 and HMECS were as in (A) above.

Based on our previous finding that FOXP3 can exert its tumour suppressive activity in part by regulating expression of miR-155 [26], we investigated whether regulation of this microRNA further contributes to the regulation of ZEB2 by FOXP3. Expression of ZEB2 is much higher in the aggressive breast cancer cell lines BT549 and MDA-MB-231, compared with its expression in normal human mammary breast epithelia (HMEC) (Figure 2A). In contrast, miR-155 expression is much higher in HMECS compared with its expression in BT549 and MDA-MB-231 cell lines (Figure 2B). FOXP3 expression is likewise higher in HMECS compared with its expression in human breast cancer cell lines (Figure 2C). Thus, FOXP3 and miR-155 expression are high in normal human breast epithelial cells (HMEC) where ZEB2 expression is low and conversely, where ZEB2 expression is high in the human breast cancer cell lines (BT549 and MDA-MB-231), FOXP3 and miR155 expression are low (Figure 2A–2C). This characterizes the system in which we propose that FOXP3 and FOXP3 induced miR-155 cooperate to inhibit ZEB2 expression to help maintain normal breast epithelial homeostasis.

Additionally, human breast cancer samples and human breast cancer cell lines have a variety of functionally significant mutations and deletions, respectively, in the FOXP3 gene [23]. FOXP3 is crucial for the function of Treg and in these cells full length FOXP3 (transcript variant 1 or Isoform a) and transcript variant 2 (isoform b) which lacks exon 3 (also known as Δ2) are both expressed [50]. These 2 isoforms of FOXP3 are likewise expressed in HMEC, whereas BT549 cells have been reported to express only transcript variant 2 which lacks exon 3 and in MDA-MB-231 cells, only FOXP3 lacking both exons 3 and 8 is expressed [23]. These deletions to the FOXP3 gene all have functional significance [23], exons 3–6 contain transcriptional repressor domains and exon 8 the leucine zipper domain. We would expect, therefore, that the BT549 and MDA-MB-231 cell lines used in this study would have one or more of these functionally significant deletions in the FOXP3 gene.

**Figure 3:**
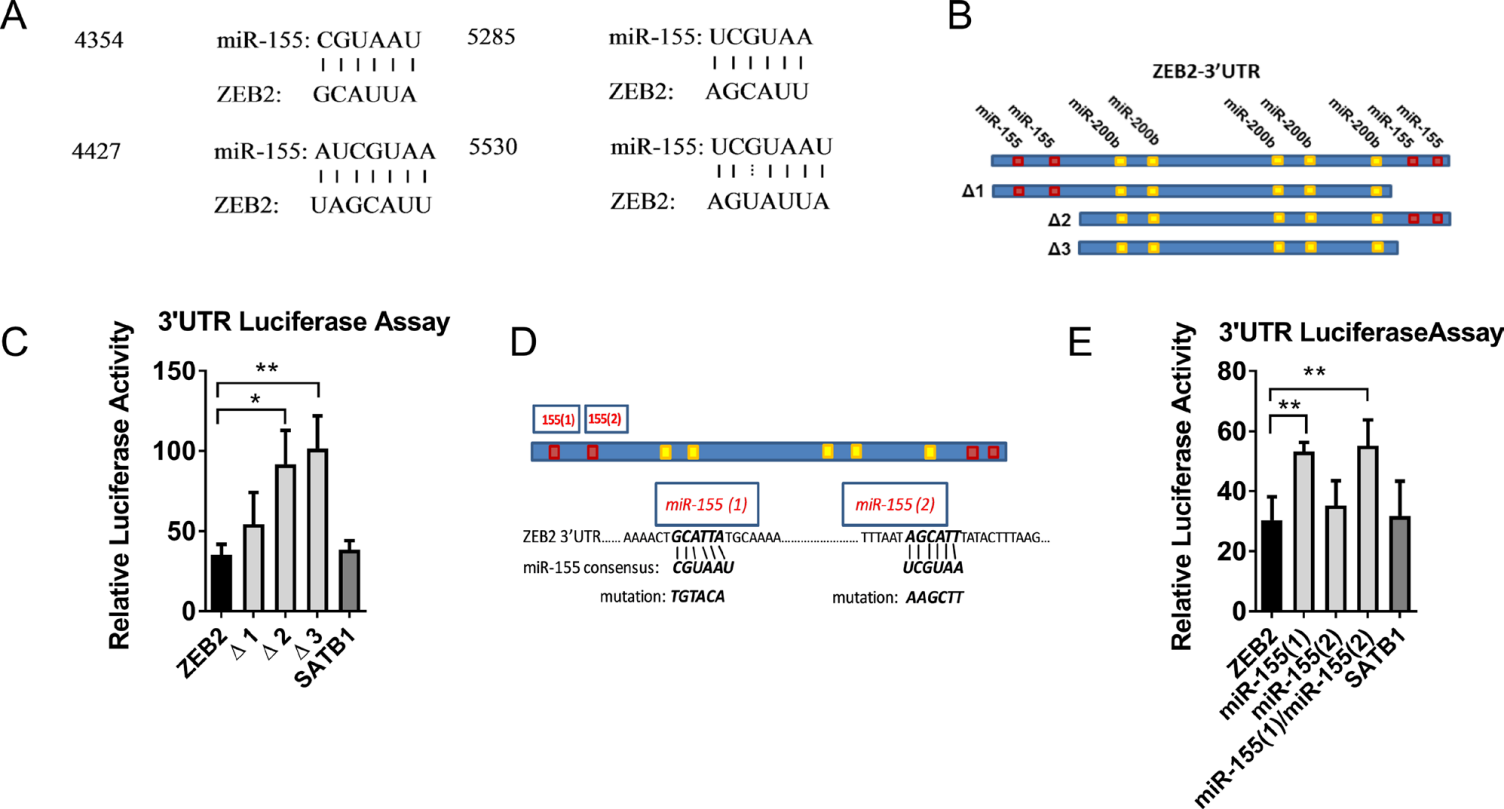
miR-155 targets ZEB2 (**A**) miR-155 and ZEB2 alignment showing miR-155 sequence and predicted alignment in the ZEB2 3′UTR (http://34.236.212.39/microrna/). (**B**) Schematic of ZEB2 3′UTR, showing location of the 4 putative miR-155 binding sites relative to known miR-200b binding sites [15]. Truncation constructs removing putative miR-155 binding sites (3) and (4) (Δ1construct), putative miR-155 binding sites (1) and (2) (Δ2 construct) and all four putative miR-155 binding sites (Δ3 construct). (**C**) Relative luciferase activity of PsiCHECK2™-ZEB2 3′UTR full length and truncation constructs Δ1, Δ2 and Δ3 in miR-155 co-transfected HEK293T cells, expressed relative to miR-control transfected cells. Ratios of reporter Renilla luciferase to normaliser Firefly luciferase activities were calculated from luminescence assays in HEK393T cells co-transfected with PsiCHECK2™-ZEB2 3′UTR (full-length or truncated) or positive control PsiCHECK2™-SATB13′UTR reporters and hsa-miR-155 or miR-Control mimics. Relative luciferase activities from cells co-transfected with miR-155 mimics were plotted normalised to cells co-transfected with miR-Control mimics. Shown is the mean + SD of three experiments. Student’s *t* test ^*^P < 0.05, ^**^P < 0.01. (**D**) Schematic of ZEB2 3′UTR showing putative miR-155 binding sites (1) and (2), miR-155 consensus sequence and introduced mutations. (**E**) Relative luciferase activity of PsiCHECK2™-ZEB2 3′UTR full length and miR-155 target sequence mutant constructs 1 and 2 (or positive control PsiCHECK2™-SATB1–3′UTR) reporters expressed relative to miR-control transfected HEK293T cells calculated as for (C) above. Shown is the mean + SD of four experiments, each with three replicate samples: Student’s *t* test ^*^P < 0.05, ^**^P < 0.01.

We identified four candidate miR-155 sites (http://34.236.212.39/microrna/) and constructed ZEB2 3′UTR reporters to test responsiveness to miR-155, and to localise miR-155 responsive regions using luciferase assays (Figure 3A, 3B). Micro RNA-155 reduced luciferase activity from the reporter containing the full ZEB2 3′UTR to 35% of its activity in miR-control treated cells (luminescence data is expressed relative to miR-control treated cells), indicating that the ZEB2 3′UTR could be directly regulated by miR-155 (Figure 3C). A comparable reduction in luciferase activity in response to miR-155 was observed with a construct containing the SATB1 3′UTR (38% of control-miR treated cells) (Figure 3C) which we previously reported as a miR-155 target [26]. In order to localise this miR-155 effect in the ZEB2 3` UTR, we made a series of truncations (Δ1, Δ 2, Δ 3) and determined that the miR-155 responsiveness of the ZEB2 3′UTR is located in the 5′end of the ZEB2 3′UTR (Figure 3C), suggesting that one or both of the miR-155 target sequences located in this region are responsible for the miR-155-mediated repression of ZEB2.

We verified that this was dependant on functional miR-155 target sequences in the ZEB2 3`UTR by disrupting each of the putative miR-155 target sequences (Figure 3D). Luciferase activity in cells co-transfected with miR-155 and the full ZEB2 3′UTR was 30% that of cells co-transfected with control-miR (Figure 3E). Luminescence was partially restored by mutation of miR-155 target site 1, but not by mutation to target site 2, suggesting that target site 1 is the predominant miR-155 target site in the 3′UTR of ZEB2 (Figure 3E). To confirm the physiological relevance of this, levels of endogenous miR-155 were reduced using miR-inhibitors in the immortalised but non-tumorigenic breast epithelial cell line, MCF10A, which expresses miR-155. ZEB2 mRNA expression was increased at 5 and 7 days post miR-155 inhibitor transfection compared with miR-control inhibitor transfected cells, indicating that endogenous miR-155 represses ZEB2. SATB1 mRNA was measured as a control target gene of miR-155 and similar findings were observed (Supplementary Figure 1A, 1B).

### miR-155 and FOXP3 cooperate to down regulate endogenous ZEB2 but not ZEB1 in breast cancer cells

**Figure 4:**
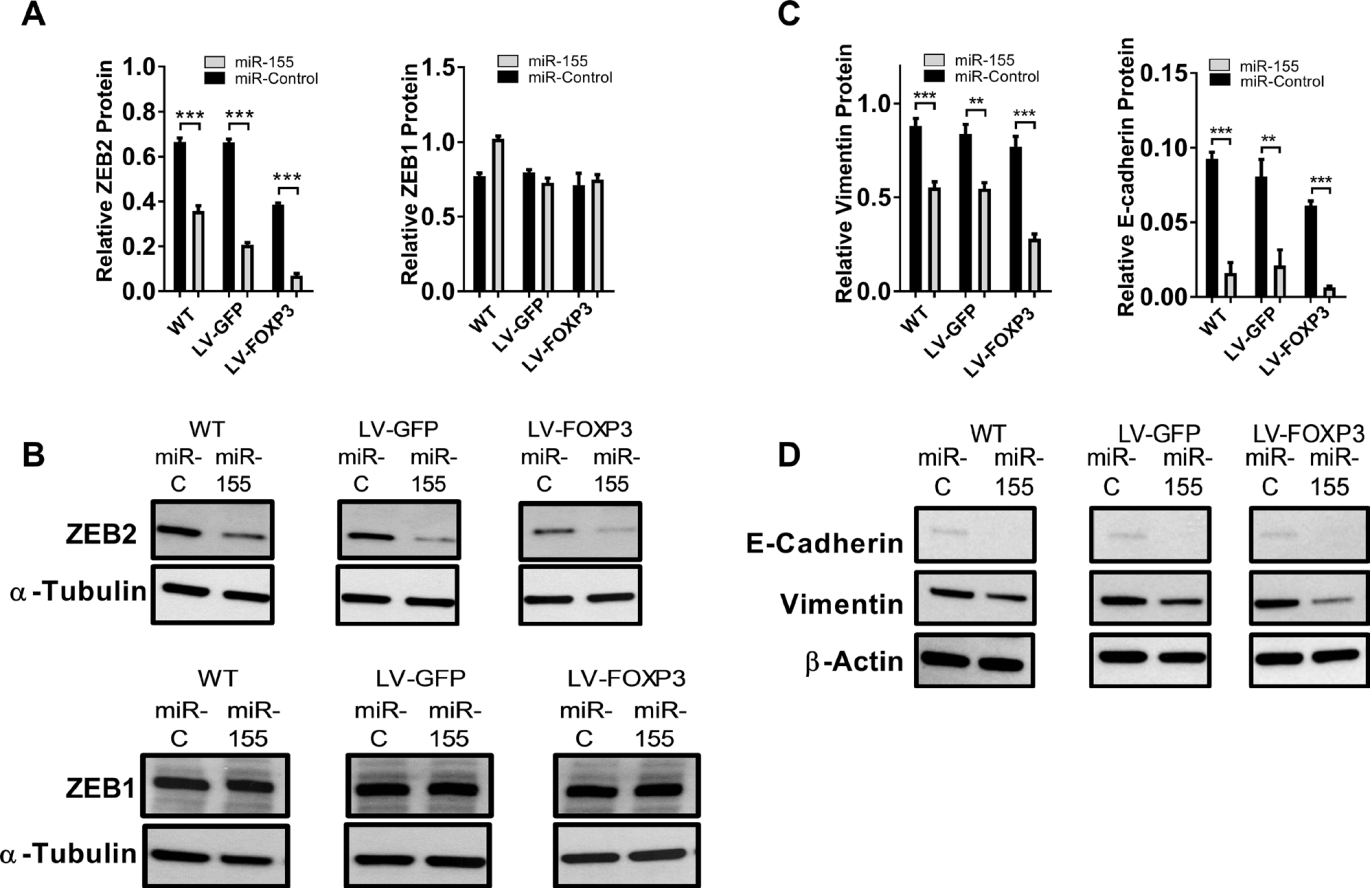
miR-155 and FOXP3 down regulate endogenous ZEB2 in human breast cancer cells resulting in altered levels of EMT markers Vimentin and E-cadherin (**A**) Relative abundance of ZEB2 and ZEB1 protein in WT, GFP or FOXP3 overexpressing BT549 cells transfected with miR-155 or miR-control. Relative abundance of protein was determined by quantitation of the abundance of ZEB2 or ZEB1 proteins normalised to reference protein α-Tubulin by western blot analysis. Quantitation of bands was carried out using Image J software. Mean + SD plotted. Student’s *t* test ^***^P < 0.001. ZEB1 protein expression as above. *n =* 3 experiments. (**B**) ZEB2 and ZEB1 protein in WT, GFP or FOXP3 overexpressing BT549 cells transfected with miR-155 or miR-control by western blot. Representative western blot shown. (**C**) Relative abundance of Vimentin and E-cadherin protein in WT, GFP or FOXP3 overexpressing BT549 cells transfected with miR-155 or miR-control. Relative abundance of protein was determined by quantitating the abundance of E-cadherin or Vimentin proteins and normalising to reference protein β-Actin by western blot analysis. Quantitation of bands was carried out using Image J software. Mean + SD plotted. Student’s *t* test ^***^P < 0.001, ^**^P < 0.01. *n =* 3 experiments. (**D**) Vimentin and E-cadherin protein in WT, GFP or FOXP3 overexpressing BT549 cells transfected with miR-155 or miR-control analysed by western blot. Representative western blot shown.

Our *in vitro* data and *in silico* predictions led us to speculate that miR-155 and FOXP3 may down regulate endogenous ZEB2 in normal breast epithelia as part of epithelial homeostasis, and this is defective in breast cancer. To test this hypothesis we overexpressed FOXP3 (or GFP control) in the breast cancer cell line BT549 and transfected these and wild- type (WT) cell lines with either miR-155 or miR-control. ZEB2 protein levels were substantially reduced, as addition of miR-155 caused a 46%, 68% and 81% decrease in ZEB2 protein in WT, GFP and FOXP3 lentiviral transduced BT549 cells, respectively (Figure 4A, 4B). The greatest reduction in ZEB2 protein levels was observed when the FOXP3 expressing cells were also transfected with miR-155 (33% lower than GFP cells transfected with miR-155) (Figure 4A, 4B). In contrast to the results with ZEB2, ZEB1 protein expression was not reduced in cells transfected with miR-155 or in FOXP3 overexpressing cells (Figure 4A, 4B). Thus, we propose that FOXP3 and miR-155 co-operate to down regulate ZEB2, but not ZEB1, in breast cancer cells.

### miR-155 and FOXP3-mediated reduction of ZEB2 expression alters Vimentin and E-cadherin

We then examined the consequences of the regulation of ZEB2 by FOXP3 and miR-155 on sentinel marker genes implicated in the invasive and migratory potential of human breast cancer cells. When we examined the expression of Vimentin, an intermediate filament protein essential for formation of invadopodia and invasion [6, 7], we observed that Vimentin protein levels were substantially reduced by miR-155 treatment (37 %, 35% and 64% reduction in WT, GFP and FOXP3 expressing cells respectively), with the greatest loss of Vimentin protein measured in miR-155 treated FOXP3 expressing cells (Figure 4C, 4D). When ZEB2 was down regulated we expected to see relief of E-cadherin repression, but interestingly we observed loss of E-cadherin in miR-155 transfected cells (83% 74% and 90% reduction in WT, GFP and FOXP3 expressing cells respectively, compared with controls) (Figure 4C, 4D). Again, the most significant decrease in E-cadherin protein was observed in FOXP3 expressing cells transfected with miR-155. These results suggest that down regulation of ZEB2 by the combination of FOXP3 and miR-155 leads to the substantial reduction of E-cadherin.

**Figure 5:**
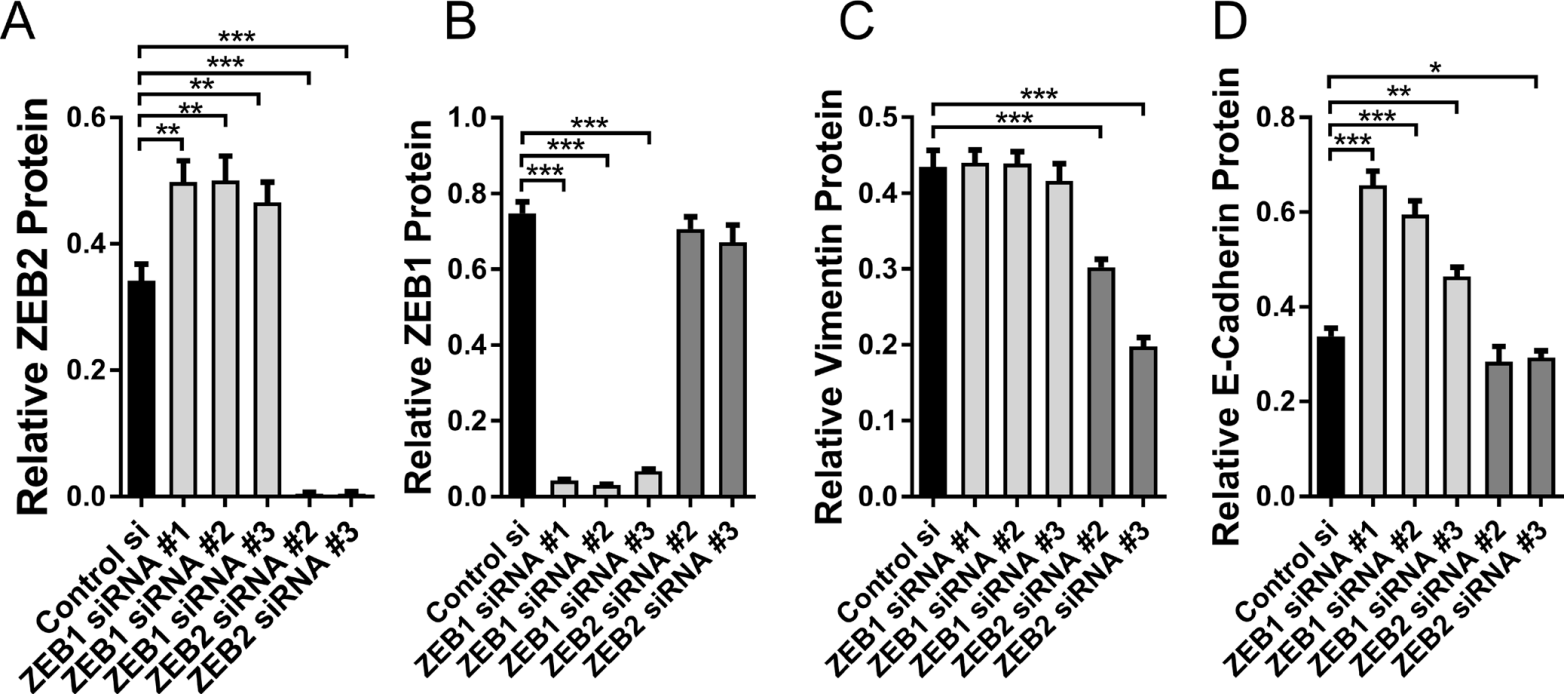
Direct targeting of ZEB2 and ZEB1 with siRNAs (**A**) Relative abundance of ZEB2 protein in BT549 cells transfected with si-Control #1, si-ZEB1#1, si-ZEB1#2, si-ZEB1#3, siZEB2#2 or si-ZEB2#3 siRNAs. Relative abundance of protein was determined by quantitating the abundance of ZEB2 protein and normalising to reference protein α-Tubulin by western blot analysis. Quantitation of bands was carried out using Image J software, mean + SD plotted. Student’s *t* test: ^**^P < 0.01, ^***^P < 0.001. *n =* 3 experiments. (**B**) Relative abundance of ZEB1 protein in BT549 cells transfected with si-Control #1, si-ZEB1#1, si-ZEB1#2, si-ZEB1#3, siZEB2#2 or si-ZEB2#3 siRNAs. Relative abundance was determined as in (A), mean + SD plotted. Student’s *t* test: ^***^P < 0.001. *n =* 3 experiments. (**C**) Relative abundance of Vimentin protein in BT549 cells transfected with si-Control #1, si-ZEB1#1, si-ZEB1#2, si-ZEB1#3, siZEB2#2 or si-ZEB2#3 siRNAs. Relative abundance of protein was determined by quantitating the abundance of Vimentin protein and normalising to reference protein β-Actin by western blot analysis. Quantitation of bands was carried out using Image J software, mean + SD plotted. Student’s *t* test: ^***^P < 0.001. *n =* 3 experiments. (**D**) Relative abundance of E-cadherin protein in BT549 cells transfected with si-Control #1, si-ZEB1#1, si-ZEB1#2, si-ZEB1#3, siZEB2#2 or si-ZEB2#3 siRNAs. Relative abundance of E-cadherin was determined as in (C), mean + SD plotted. Student’s *t* test: ^*^P < 0.05, ^**^P < 0.01, ^***^P < 0.001. *n =* 3 experiments.

Neither E-cadherin nor Vimentin contain miR-155 seed sequences and were not identified as potential FOXP3 target genes in our Treg FOXP3 ChIP dataset [31]. However, to confirm that changes in Vimentin and E-cadherin expression were via direct regulation by ZEB2 and not by direct interactions with FOXP3 or miR-155, we then depleted the breast cancer cells of ZEB1 or ZEB2 using siRNAs. We tested several different siRNAs to each of ZEB1 and ZEB2 and to validate their specificity, we measured the expression of ZEB2 and ZEB1 protein in cells treated with the siRNAs for both ZEB1 and ZEB2. ZEB2 si#2 and si#3 gave on-target knockdown of ZEB2 but not ZEB1 (Figure 5A, 5B). Interestingly, when we tested ZEB2 siRNA si#1 we observed substantial off- target effects (not shown), so this si-RNA was not used in further studies. ZEB1siRNAs si#1 si#2, and si#3 all gave specific on- target knockdown of ZEB1 (Figure 5B). Interestingly, the siZEB1 siRNAs resulted in substantially increased ZEB2, possibly because ZEB1 either directly or indirectly imposes restraint on ZEB2 expression (Figure 5A). si-ZEB1 did not change Vimentin expression whilst siZEB2 led to reduced Vimentin expression (30% less, 54% less than control si-RNA with siZEB2 #2 and #3 respectively) (Figure 5C), confirming that ZEB2, but not ZEB1, regulates Vimentin expression. Si-ZEB1 resulted in de-repression of E-cadherin protein (50%, 43%, 27% higher than control si-RNA for siZEB1 #1, 2, 3 respectively), as expected, and consistent with ZEB1 down regulating E-cadherin (Figure 5D). Since depleting ZEB1 resulted not only in increased E-cadherin but also increased ZEB2, it seems unlikely, therefore, that ZEB2 would inhibit E-cadherin expression. This is supported by the finding that depletion of ZEB2, but not ZEB1, using si-ZEB2, did not de-repress E-cadherin, and it is either unchanged or further reduced by this treatment (siZEB2#2 n/s change, siZEB2 #3 15% lower than control si-RNA) (Figure 5D). Although ZEB2 siRNAs resulted in almost total depletion of ZEB2 (Figure 5A) we did not see the substantial loss of E-cadherin as observed when ZEB2 was downregulated by miR-155 and FOXP3 (Figure 4C, 4D). As mentioned above, although E-cadherin is not predicted to be a target of either miR-155 or FOXP3 directly, from this result we cannot preclude the possibility that there may have been an indirect effect on E-cadherin. This is not surprising given that FOXP3 is a transcription factor that orchestrates networks of interactions and is a known tumour suppressor in breast cancer, and miR-155 has been shown to have a complex role in the breast epithelium: maintaining normal breast epithelial homeostasis and also playing a role in breast cancer. However, our results do show that whereas depletion of ZEB1 using siRNAs resulted in de-repression of E-cadherin, this was not the case when ZEB2 was depleted using siRNAs, indicating that ZEB2 does not repress expression of E-cadherin., Recently published ZEB2 chip-Seq data, using human cancer cell lines, indicated that the *CDH1* gene (E-cadherin) was not a target of ZEB2, it was not enriched by ChIP and furthermore E-cadherin expression was not increased by depletion of ZEB2, thus reinforcing our conclusions [51].

### miR-155 and FOXP3-mediated reduction of ZEB2 expression alters invasion and migration

**Figure 6:**
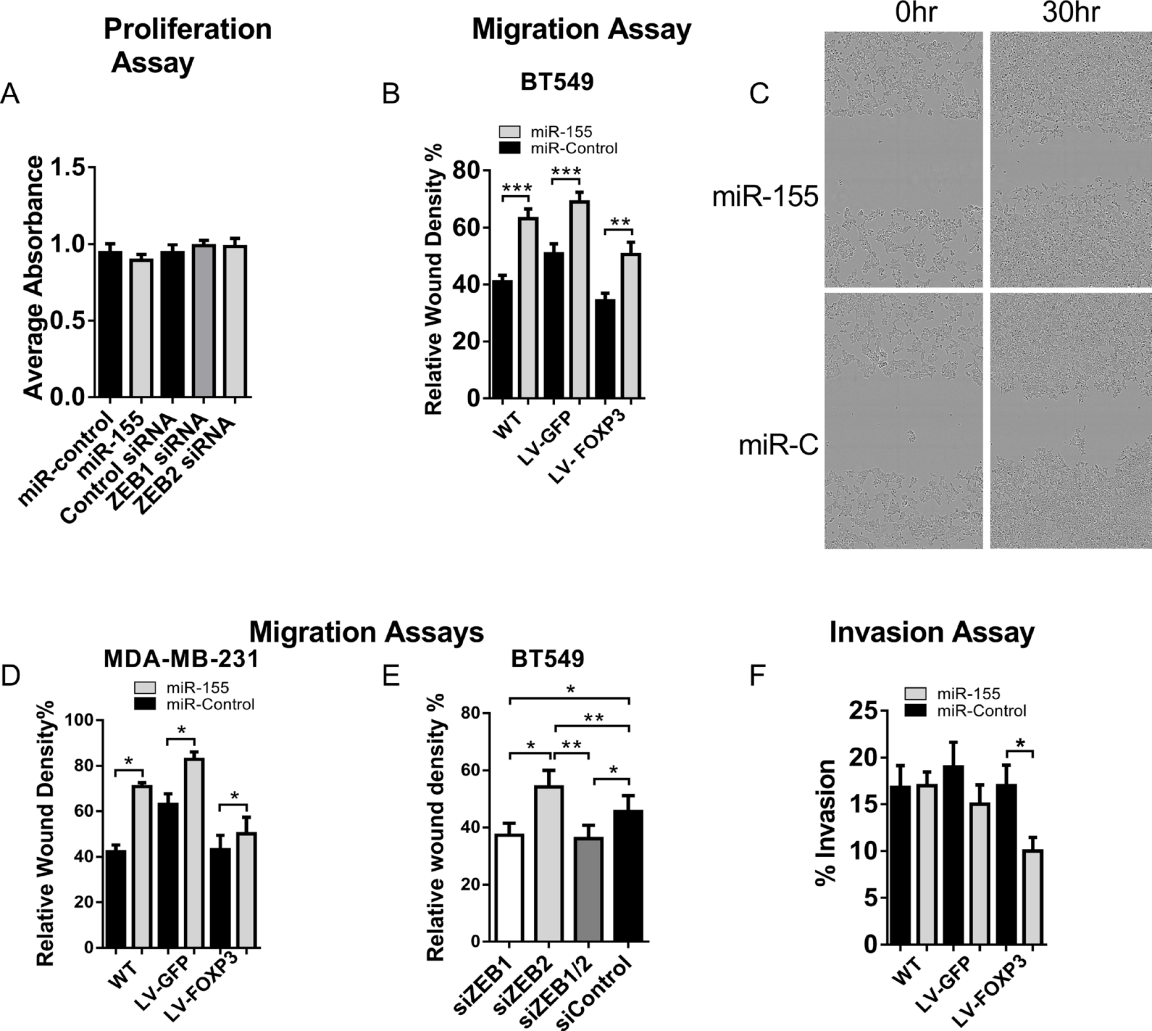
Loss of ZEB2 leads to altered migration and invasion in human breast cancer cells (**A**) Proliferation in miR-control, miR-155, si-Control, si-ZEB1 or si-ZEB2, transfected BT549 cells. Proliferation was measured by colorimetric analysis (CellTiter 96^®^ Aqueous One solution Cell Proliferation Assay kit, Promega). The quantity of Formazan product measured by absorbance at 490 nm is directly proportional to the number of living cells. Absorbance of 4 technical repeats (no cell control absorbance deducted) to generate a corrected absorbance and means + SD were plotted. *n =* 3 experiments. (**B**) Real-time cell migration in WT, GFP or FOXP3 overexpressing BT549 cells transfected with miR-155 or miR-control. Relative Wound Density (%) was calculated using the custom algorithms supplied with the IncuCyte™ software. Cell type specific Processing Definition Algorithms were used to analyse the data (see Methods). Relative Wound Density data was generated for 4 technical replicates over the 50 hour period (see Supplementary Figure 2) for each experiment. The means + SEM of Relative Wound Density data collected at 40 hours (minus 0 hour data) for 6 experiments is plotted. Student’s *t* test: ^***^P < 0.001, ^**^P < 0.01. (**C**) Representative Images of BT549 cells transfected with miR-155 or miR- Control (miR-C) showing initial scratch wounds at 0 hours and 30 hours thereafter. (**D**) Real-time cell migration in WT, GFP or FOXP3 overexpressing MDA-MB-231 cells transfected with miR-155 or miR-control. Relative Wound Density (%) was calculated as described in B above. Relative Wound Density data was generated for 4 technical replicates over the 50 hour period for each experiment. The means + SEM of Relative Wound Density data collected at 30 hours (minus 0 hour data) for 3 experiments is plotted. Student’s *t* test: ^*^P < 0.05. (**E**) Real-time cell migration in WT-BT549 cells transfected with si-Control, si-ZEB2, si-ZEB1 or si-ZEB1 and si-ZEB2 siRNAs together. Relative Wound Density (%) was calculated as described in B above. Relative Wound Density data was generated for 4 technical replicates over the 50 hour period for each experiment. The means + SEM of Relative Wound Density data collected at 40 hours (minus 0 hour data) for 4 experiments is plotted. Student’s *t* test: ^*^ *P <* 0.05, ^**^P < 0.01. (**F**) Percentage invasion of WT, GFP or FOXP3 over-expressing BT549 cells transfected with miR-155 or miR-control. Transfected cells were serum starved in 0.5% FCS for 24 hours prior to analysis of invasion. Cells (1 × 10^4^) were then seeded (with 3 technical replicates) onto BME coated invasion chambers. Invasion assays were carried out according to manufacturer’s instructions (Cultrex^®^ Cell Invasion Assay) and fluorescence was read at 485 (Excitation) and 520 nm (Emission). Background fluorescence was subtracted from the Relative Fluorescence Units (RFU) obtained and these were converted to cell number using a standard curve generated for each experiment. Percentage Cell Invasion was calculated according to the Cultrex^®^ Cell Invasion Assay instructions. Mean % cell invasion of a representative experiment + SEM of 4 experiments is shown. Student’s *t* test: ^*^ *P <* 0.05.

In order to demonstrate a link between gene expression and function, we next investigated the functional effect of ZEB2 loss on migration and invasion. Using siRNAs to ZEB1 and ZEB2 and miR-155 or controls, we first confirmed that neither increased miR-155 nor specific loss of ZEB2 or ZEB1 altered the proliferation of transfected BT549 cells (Figure 6A). A scratch wound assay using an IncuCyteZOOM™ was then used to model real time migration. Cell migration data was collected over a 50 hour time period and analysed using the Relative Wound Density software algorithms supplied. The relative wound density metric is the most accurate metric to assess cell migration. We found that in WT, GFP and FOXP3 expressing BT549 cells transfected with miR-155, migration was increased compared with control cells (Supplementary Figure 2A–2C; single experiment with 4 technical replicates). We used miR200b as a control since miR-200b downregulates both ZEB1 and ZEB2, and in our initial experiments we compared migration in miR-200b transfected BT549 cells with cells transfected with miR-155 or miR-control. In the miR-200b transfected cells there was very low cell migration (Supplementary Figure 2A–2C). We then collated the analysed migration data at a fixed time point of 40 hours from 6 experiments and this showed that migration was increased in WT, GFP and FOXP3 expressing BT549 cells transfected with miR-155 (35%, 26.5% and 32% increase respectively) relative to miR-control transfected cells (Figure 6B), indicating that loss of ZEB2 resulted in increased migration. A representative set of images of BT549 cells transfected with miR-155 or miR- Control (miR-C) shows initial scratch wounds at 0 hours and 30 hours thereafter (Figure 6C). Similar results were observed using a second highly invasive breast cancer cell line MDA-MB-231; cells transfected with miR-155 relative to miR-control transfected cells (40%, 24%, 14% increased migration in WT, GFP and FOXP3 cells respectively; Figure 6D). We then depleted ZEB2 in cells using siRNAs to determine whether migration was enhanced by direct depletion of ZEB2. We observed significantly increased migration in cells transfected with si-ZEB2 compared with si-Control (18% higher than si-Control) (Figure 6E). In contrast, cells transfected with si-ZEB1 had reduced migration compared with si-Control (18% lower than si-Control), and this is linked to the de-repression of E-cadherin and more adherent properties. The results from the siZEB1 siRNA experiments and the conclusions drawn are consistent with previous studies [52]. Migration in cells transfected with si-ZEB2 was significantly higher than in cells transfected with si-ZEB1 (32% higher) and interestingly, migration was decreased in cells transfected with si-ZEB1 and si-ZEB2 together compared with si-Control (21% lower) and also compared with cells transfected with si-ZEB2 alone (34% lower) (Figure 6E). Similar results were obtained using MDA-MB-231 cells (data not shown). These results provide a functional link with our findings that E-cadherin is not de-repressed when ZEB2 is depleted and as a result of this E-cadherin levels are low (Figure 5D). Cells are therefore less adherent than cells depleted of ZEB1 and therefore the migratory potential of cells with depleted ZEB2 is higher than in cells with depleted ZEB1. Since Vimentin expression is linked to the invasive potential of cells, we next examined the contribution of FOXP3 and miR-155 mediated ZEB2 loss to changes in cell invasion. Invasion was reduced by 40% in FOXP3 expressing BT549 cells treated with miR-155 relative to miR-control treated cells (Figure 6F). We suggest that reduced Vimentin expression resulting from the dual FOXP3 and miR-155 dependent down regulation of ZEB2, impairs invadopodia formation and this reduces invasion. We conclude that down regulation of ZEB2 by miR-155 and FOXP3 alters the migratory and invasive potential of breast cancer cells.

### FOXP3 regulates ZEB2 expression in normal human mammary epithelial cells

**Figure 7:**
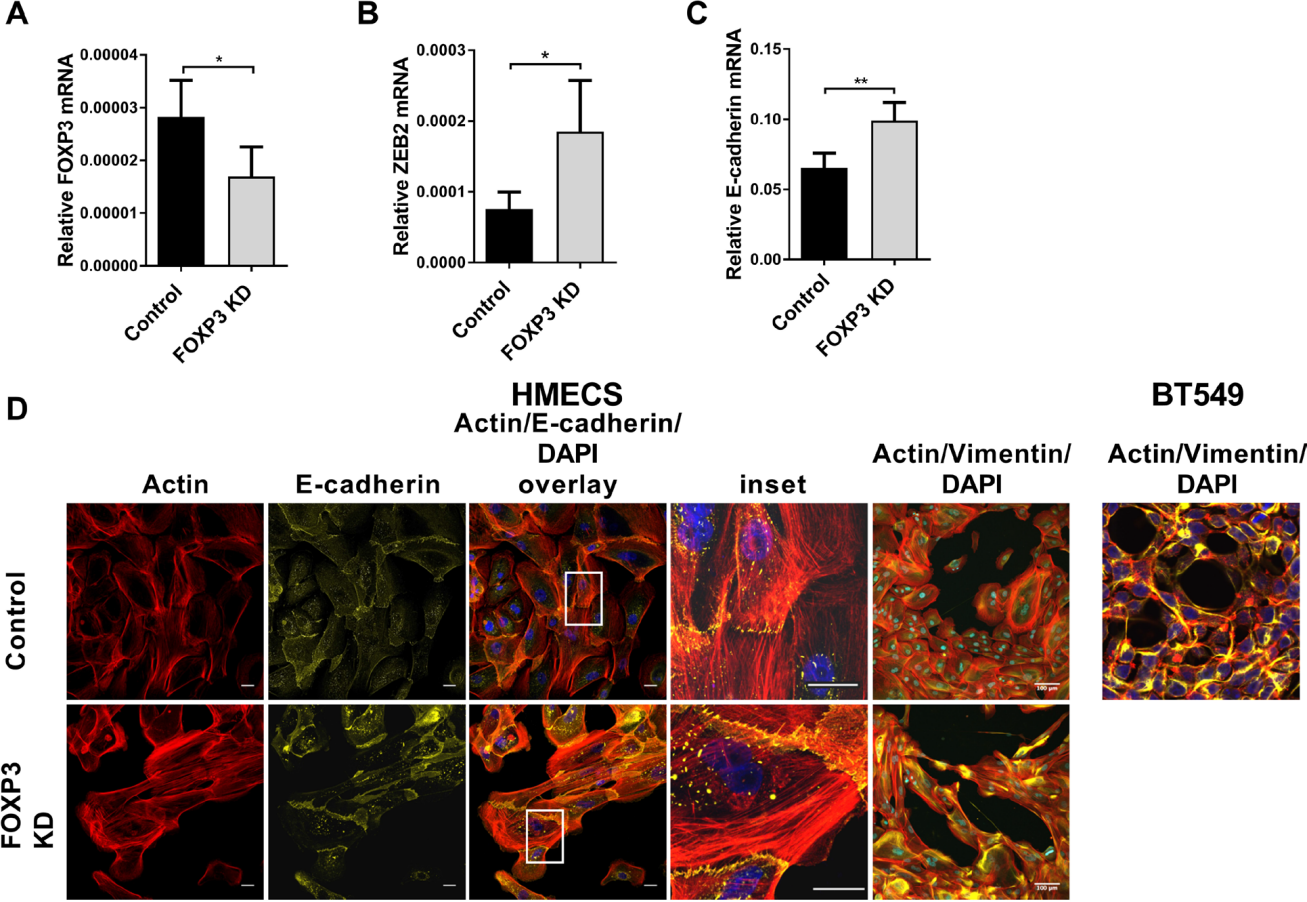
FOXP3 regulates ZEB2 expression in normal human mammary epithelial cells (**A**) Normal human mammary epithelial cells (HMEC) (passage 2) were transduced with either LV-FOXP3 KD or LV-Control lentivirus at MOI = 1 in the presence of 8ug/ml Polybrene. 72 hr after transduction total mRNA was isolated. Relative abundance of FOXP3 mRNA normalised to reference gene RPL13A mean + SEM is plotted, Student’s *t* test ^*^P < 0.05. Quantitative real-time PCR reactions were in triplicate and the means of the threshold cycles (Cts) were used for quantitation. A standard curve to determine amplification efficiency was generated for FOXP3 and for the reference gene RPL13a mRNAs (see Methods section). The standard curve method for relative quantitation was used to determine the relative abundance of FOXP3 mRNA normalised to the RPL13a reference gene. *n =* 4 experiments. (**B**) Normal human mammary epithelial cells (HMEC) were transduced as above. Relative abundance of ZEB2 mRNA normalised to reference gene RPL13A is plotted + SEM, Student’s *t* test, ^*^P < 0.05. Reactions for quantitative real -time PCR were run in triplicate, standard curves to determine amplification efficiency were determined for ZEB2 and RPL13a reference and the standard curve method for determination of relative abundance of mRNA was as in (A) above. *n =* 4 experiments. (**C**) Normal human mammary epithelial cells (HMEC) were transduced as above. Relative abundance of E-cadherin mRNA normalised to reference gene RPL13A is plotted + SEM, Student’s *t* test, ^**^P < 0.01. Reactions for quantitative real -time PCR were run in triplicate, standard curves to determine amplification efficiency were determined for E-cadherin and RPL13a reference and the standard curve method for determination of relative abundance of mRNA was as in (A) above. *n =* 4 experiments. (**D**) HMEC cells were transduced with LV-FOXP3 KD or LV-control at an MOI = 1. 72 hr after virus transduction, cells were processed for immunofluorescence analysis: Alexa Fluor 647 conjugated – anti-E-cadherin (1:20) or AlexaFluor^®^ 647conjugated anti-vimentin (1:50). Samples were counter stained with Alexa Fluor^®^ 568 Phalloidin (F-Actin probe) and mounted in ProLong^®^ Gold Antifade Fluorescent reagent, containing (DAPI) for nuclear staining. Confocal images were collected using identical exposure settings for each antibody fluorophore for FOXP3 KD and Control lentivirus transduced cells. Images are compositions of 12 optical sections of a Z-stack. Bar = 100 um. Inset shows enlargement of boxed area. BT549 cells similarly cultured and treated for comparison.

We then extended our studies to examine ZEB2 regulation by FOXP3 in normal human mammary epithelial cells (HMECS). Cells were transduced with shRNAi lentivirus to knockdown expression of endogenous FOXP3 [53], or control lentivirus. Gene expression and morphological changes were observed. FOXP3 knockdown (37% lower than control, Figure 7A) de-repressed ZEB2 expression (2.4× higher than control, Figure 7B) and consistent with our earlier experiments, we observed a ZEB2-induced increase in E-cadherin expression (34% higher than control; Figure 7C). Morphology of the FOXP3 knockdown HMEC cells was different from the control cells: whereas control cells were relatively small and had the regular cobblestone appearance characteristic of epithelial cells, the FOXP3 knockdown HMECS were much larger and had a disorganised, irregular appearance. They did not quite have the classical elongated appearance characteristic of cells undergoing epithelial to mesenchymal transition, although they were clearly different from the control transduced cells. Vimentin staining and also filamentous actin (F-Actin) staining were much more intense and widespread in the FOXP3 knockdown cells, with the Vimentin staining in these cells resembling that of highly invasive BT549 breast cancer cells (Figure 7D). Consistent with our gene expression data (Figure 7C) up-regulated E-cadherin staining was observed in the FOXP3 knockdown HMECS compared with control HMECS (Figure 7D). Upregulated E-cadherin was particularly observed at cell junctions, a feature consistent with greater adherence of cells. In contrast, the increased expression of actin stress fibres is generally associated with increased cell migration [54–56] and whilst increased Vimentin is generally associated with the ability to form invadopodia and thus promote invasive capacities during EMT [7], it can also promote cell motility [55]. This aberrant expression of E-cadherin combined with increased expression of F-actin and Vimentin perhaps gives rise to the unusual cell morphology observed whereby adherent cells are becoming hyperplastic. The results we present show that FOXP3 controls ZEB2 expression in normal human mammary epithelial cells (HMECS) and when FOXP3 is depleted, endogenous ZEB2 expression is de-repressed leading to dramatic changes in cell morphology as well as gene expression.

## DISCUSSION

FOXP3 has been previously identified as a tumour suppressor by our lab [26] and others (23–25, 27, 28, 30). The physiological importance of this role is suggested by the observation that up to 80% of cancers have some form of loss of function of FOXP3, and this includes loss of FOXP3 protein, loss of the full length transcript and loss of nuclear localisation, each of which results in loss of transcriptional regulation capacity by FOXP3 [23]. FOXP3 can act both directly, as a transcriptional repressor, and indirectly, by inducing a set of microRNAs that can target the same genes. This generates feed forward loops which tightly control gene expression. We previously confirmed the importance of this feed forward mechanism by demonstrating that FOXP3 and miR-155 (a microRNA up regulated by FOXP3) together tightly control SATB1 levels in both breast epithelial cells [26] and in Treg [35]. However, other targets of FOXP3 and microRNA feed forward loops remain largely unknown. Expression of FOXP3 and miR-155 are normally high in healthy breast epithelia, but during breast cancer progression FOXP3 is either aberrantly localised to the cytoplasm or is lost [23, 30]. Using our Treg FOXP3 ChIP dataset [31] we identified ZEB2 as a potential FOXP3 target gene whose 3′UTR also contained putative miR-binding sites for FOXP3 regulated miRs. ZEB2 has a prominent role in driving epithelial to mesenchymal transition (EMT) in development, fibrosis and in cancer, where high levels correlate with many types of invasive cancer, including the most malignant form of breast cancer; the triple negative basal- like carcinoma [1, 8, 9]. Thus, tight control of ZEB2 is critical for normal tissue homeostasis. We therefore hypothesised that ZEB2 could also be regulated by both FOXP3 and FOXP3 controlled microRNAs in human breast epithelial cells. Few direct transcriptional regulators of ZEB1 or ZEB2 have been identified [9], although recently ZEB1 has been shown to be a direct transcriptional target of FOXC2 [14] and ZEB2 a direct transcriptional target of T-bet [18]. Here we show that FOXP3 directly regulates the transcription of ZEB2 by binding to a site in intron 2 [31]. This region was identified in ENCODE as enriched for histone modifications which are associated with regulatory activity (Figure 1A). To investigate the possibility of dual regulation by FOXP3 and miRs we screened ZEB2 for binding sites of FOXP3 regulated miRs, and this revealed that miR-155 was a strong candidate as 4 sites were identified. All four of the putative miR-155 sites we identified in the ZEB2 3′UTR were imperfect matches to the miR-155 “seed” region (6/8 nucleotides) and interestingly, we determined that most of the functional activity is through just one of these sites. This site was independently identified as a target of miR-155 in mouse Treg using dCLIP [57], revealing conservation between species. We observed that FOXP3 and miR-155 expression are low in breast cancer cells compared with their expression in normal human breast epithelium and since we show that miR-155 can suppress ZEB2 in human breast cancer cells, we suggest that ZEB2 is tightly regulated by a FOXP3/miR-155 feed forward loop in healthy breast epithelium. MicroRNA-155 has been widely reported as playing a role in promoting many cancers, including breast cancer, leading to its being described as an “oncomir” [40–45]. However, it has also been reported as having a protective role in cancer [46–48] suggesting that its role in normal breast epithelia as well as in breast cancer is more complex. It may have multiple roles, helping to maintain homeostasis in normal breast epithelium, but also playing a changing role during different phases of cancer metastasis. Hence, its activity may be dependent on a number of different factors, including global changes in gene expression, the loss of master regulators such as FOXP3, or the accumulation of other mutations in the cell. These findings are not incompatible with one another; key genes will often have multiple layers of regulation to ensure flexibility of responsiveness.

The co-regulation of ZEB1 and ZEB2 by post-transcriptional regulatory mechanisms, such as by the miR-200 family [15–17] is well documented. However, while there are some studies showing independent regulation of ZEB2 [58] or ZEB1 [14, 59] alone, by post-transcriptional means, they did not address the impact of manipulation of one ZEB family member on the other. Furthermore, none of the published studies have investigated a role for FOXP3/miRs in control of these genes. Therefore, by defining a mechanism for the regulation of ZEB2 independently of ZEB1, which is mediated by FOXP3 and miR-155, we have been able to analyse the specific contribution of ZEB2 in human breast cancer cells.

To investigate the biological consequences of the differential regulation of ZEB2 we assayed documented targets of ZEB2 including E-cadherin [4] and Vimentin [5]. E-cadherin is required for cell adhesion [1, 8, 9] and its loss in breast cancer correlates with increased migration, a hallmark of EMT. In contrast, expression of Vimentin, an intermediate microfilament protein required for formation of invadopodia, the actin-rich membrane protrusions required for remodelling extra cellular matrix [6] and required for invasion [7], increases during EMT. Importantly, neither E-cadherin nor Vimentin contain miR-155 seed sequences and were not identified as potential FOXP3 target genes in our Treg FOXP3 ChIP dataset [31]. In our experiments, down regulation of ZEB2, surprisingly, did not de-repress E-cadherin and we observed enhanced migration. Direct down regulation of ZEB1 or ZEB2 using siRNAs confirmed these results and lead us to suggest that ZEB2 may not participate in the down regulation of E-cadherin in human breast cancer cell lines, in agreement with the finding of Caramel *et al.* in melanoma [12], but contrasting with earlier reports suggesting direct down regulation of E-cadherin by ZEB2 alone [4]. Other studies using siRNA to target ZEB1and ZEB2 have led to differing findings: Park *et al.* [17] concluded that both ZEB family members were required for E-cadherin repression and also observed cross reactivity with siRNAs to ZEB1 and ZEB2, whilst other studies using either siRNA pools [15] or siRNA to both family members [16] concluded that each of ZEB1 and ZEB2 participate in the down regulation of E-cadherin, but it may have been unappreciated in these papers that both ZEB1 and ZEB2 were simultaneously targeted [15, 16]. Moreover, recently published ZEB2 chip-Seq data, using human cancer cell lines, indicated that *CDH1* (the E-cadherin gene) was not a target of ZEB2, it was not enriched by ChIP and furthermore, E-cadherin expression was not increased by depletion of ZEB2 [51]. We find that ZEB2, but not ZEB1, is required for Vimentin expression, since specific targeting of ZEB1 using siRNA did not alter Vimentin expression, whereas Vimentin expression was abrogated when ZEB2 was down regulated by FOXP3, miR-155 and also by ZEB2 siRNAs. This is in agreement with previous reports indicating Vimentin expression is increased by ZEB2 [5]. Consistent with this, we also observed reduced invasion *in vitro*, although only in cells with the most depleted ZEB2 (FOXP3 expressing cells transfected with miR-155) presumably because Vimentin protein has to be sufficiently depleted before invasive capabilities are compromised. Hence, ZEB2 is specifically required for invasive capacity during cancer metastasis.

We have also shown that in normal cultured human breast epithelial cells (HMECS) FOXP3, and by inference miR-155, control ZEB2 expression, and this helps to maintain a normal breast epithelial phenotype. When FOXP3 was depleted, ZEB2 was de-repressed. The de-repression of ZEB2 resulted in dramatic changes to the breast epithelial cells. The cell morphology was quite altered: whereas the control cells had the classical, small, cobblestone appearance characteristic of epithelial cells, the FOXP3 depleted cells were much larger, irregularly shaped and elongated, although they had not acquired the classical spindle shape characteristic of mesenchymal cells. We observed increased Vimentin (a classical marker of EMT associated with acquisition of invasive properties), consistent with cells forming invadopodia to allow degradation of the extra-cellular matrix. Vimentin however, has a complex role in the cell. It can promote cell motility even in the presence of E-cadherin [55] and in migrating cells intermediate filament proteins, including Vimentin, undergo massive reorganisation, associating with a network of cytoskeletal components including actin filaments. Actin filaments are integral to cytoskeletal function, controlling cell polarity, generating traction forces and controlling extra-cellular matrix remodelling, to achieve directional migration [54] and thus the increased F-actin we observed is consistent with more motile cells. Further analysis of Vimentin and F-actin distribution in the cell was, however, beyond the scope of this study. We observed aberrant enhancement of E-cadherin expression, especially along cell junctions, suggesting increased and not decreased cell adhesion. These findings in HMECS support our breast cancer cell data, in which we suggest that ZEB2 does not down regulate E-cadherin. The aberrant E-cadherin expression we observed in FOXP3 knockdown HMECs may explain the unusual morphology of the cells, whereby increased F-actin and Vimentin promote cytoskeletal rearrangement consistent with an invasive phenotype, whereas the increased E-cadherin supports cell-cell adherence. We speculate that these unusual characteristics are the result of disrupting part of the EMT process by manipulation of ZEB2 expression, allowing the cells to become hyperplastic without undergoing full EMT. However, it is normal for these processes to be quite dynamic, as cells can alternate between stationary or migratory modes [54].

**Figure 8:**
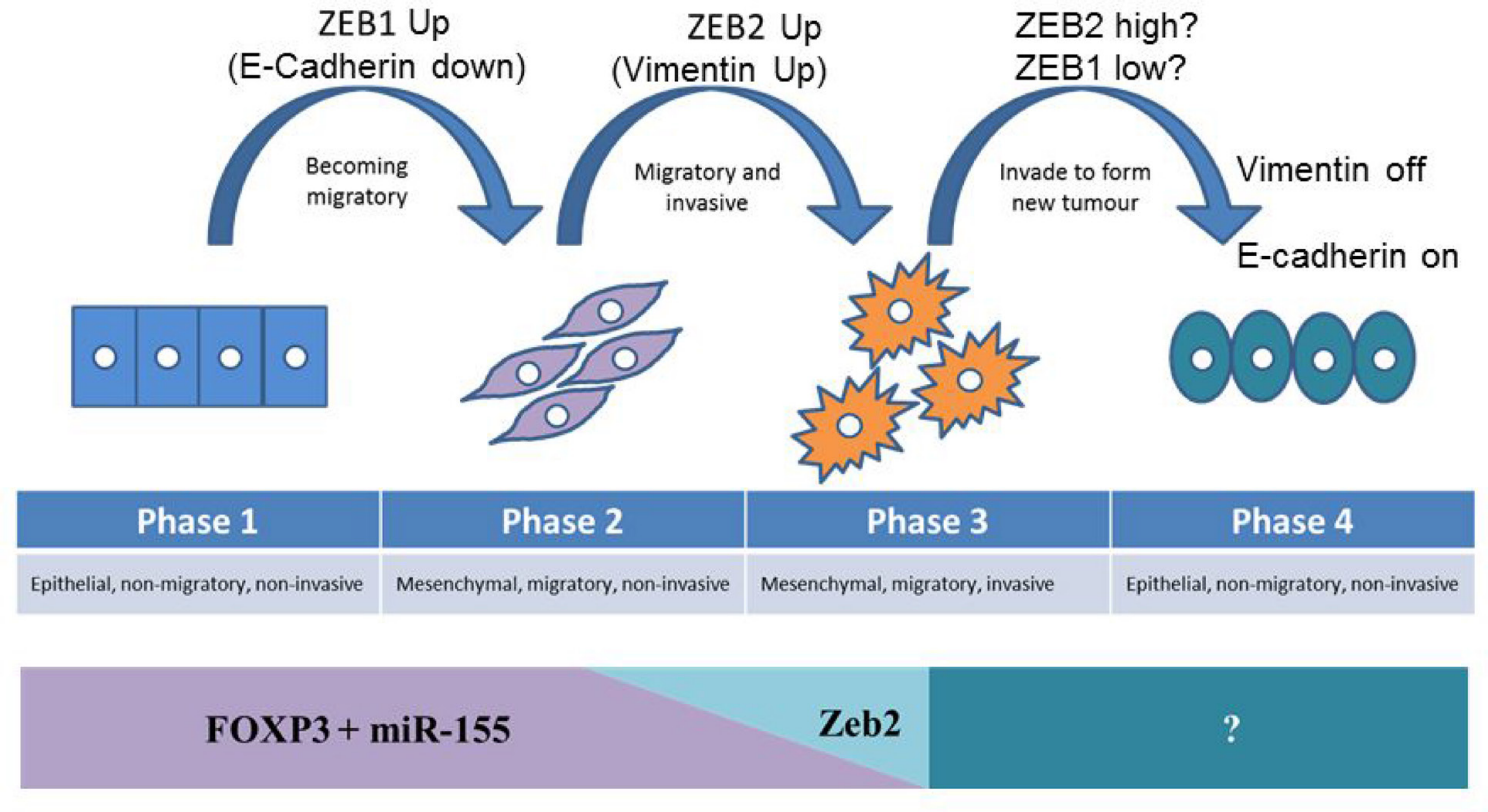
Model of the contribution of FOXP3 mediated repression of ZEB2 to the prevention of progression of metastatic potential, and the impact of loss of FOXP3 on metastasis In healthy epithelium the tumour suppressor FOXP3 restrains oncogenes, including ZEB2, but does not control ZEB1. Phase 1: loss of repression of ZEB1 (by other mechanisms) is an early step in transformation, and this decreases the expression of E-cadherin, facilitating migration. Phase 2: If loss of FOXP3 then occurs, ZEB2 is no longer controlled, resulting in up regulation of Vimentin, leading to degradation of the extra-cellular matrix, facilitating invasion. Phase 3: In order for migratory and invasive (metastatic) cells to form a solid tumour at a new site, adherent properties need to be re-established, but as metastasis results in solid tumours at multiple sites simultaneously, these transitions are dynamic. If E-cadherin needs to be upregulated, this could be facilitated by loss of ZEB1 expression, leaving ZEB2 unaltered. Phase 4: Establishing a secondary tumour at the new site requires de-repression of E-cadherin to facilitate cell adhesion, and reduced Vimentin to curtail invasive capabilities, as a result of inhibition of ZEB2, leading to a stable secondary tumour.

Maintenance of epithelial integrity involves multiple cell-cell adhesion complexes and cytoskeletal rearrangements and since ZEB2 has been shown to regulate other tight junction proteins [60], it is quite likely therefore, that FOXP3 and ZEB2, directly or indirectly, control waves of gene expression to create complex networks of protein interactions which help shape the process of EMT (and metastasis). This gives a new insight into the role of ZEB2 and also perhaps of ZEB1 in breast cancer metastasis (Figure 8). It is conceivable that the steady state expression of the tumour suppressor FOXP3 in healthy epithelium restrains oncogenes, including ZEB2, but cannot control ZEB1, so that loss of repression of ZEB1 (by other mechanisms) is an early step in transformation, and this decreases the expression of E-cadherin, facilitating migration (Figure 8 phase 1–2). If loss of FOXP3 then occurs, ZEB2 is no longer controlled, resulting in up regulation of Vimentin, leading to degradation of the extra-cellular matrix facilitating invasion (Figure 8 phase 2–3). In order for migratory and invasive (metastatic) cells to form a solid tumour at a new site, adherent properties need to be established. If E-cadherin needs to be upregulated, this could be facilitated by loss of ZEB1 expression, leaving ZEB2 unaltered (Figure 8 phase 3–4). This model is based on the observations from Figure 7 where removal of FOXP3 in HMEC resulted in both upregulation of Vimentin, and E-Cadherin. At the new tumour site de-repressed E-cadherin facilitates cell adhesion, and then inhibition of ZEB2, leads to reduced Vimentin and lowered invasive capabilities resulting in a stable secondary tumour. This model is necessarily simplified as it does not allow for the complexity of the EMT process, or multiple functions of E-Cadherin and Vimentin, but it may illustrate the contribution of FOXP3 and FOXP3 induced miR-155 in maintaining breast epithelial homeostasis. In conclusion, we speculate that ZEB2 and ZEB1 may have subtly different roles in breast cancer metastasis and that the current models of cancer progression may not reflect the complexity and hierarchy of the network of interactions that orchestrate these programmes.

## MATERIALS AND METHODS

### Cell culture

HEK293T and the breast carcinoma cell lines BT549 and MDA-MB-231, were from the ATCC repository, thawed and cultured accordingly for fewer than 15 passages. Primary human epithelial mammary cells (HMEC) were purchased (CC-2551, Lonza, Australia), thawed and cultured using Mammary Epithelia Growth Medium and Bullet Kit™ (CC-3150, Lonza, Australia) and ReagentPack™ (CC-5034, Lonza, Australia) for fewer than 4 passages. MCF10A cells were purchased from ATCC^®^ repository (CRL-10317), thawed and cultured for fewer than 10 passages in 1:1 DMEM/Ham’s F12 (Sigma 51651C), 5% Horse serum (Sigma H1270), 20 ng/ml EGF (Sigma E9644), 0.5 ug/ml Hydrocortisone (Sigma H0888), 100 ng/ml Cholera Toxin (Sigma C8052), 0.25 U/ml Insulin (Protaphane Penfill, Novo Nordisk Pharmaceuticals PTY. LTD, NSW, Australia).

### FOXP3 ChIP assays

FOXP3 ChIP assays were performed as before [31] on BT549 cells transiently transfected with the FOXP3 expression vector pLV411-FOXP3 [53]. ChIP assays were carried out with either a rabbit anti-Foxp3 IgG (Novus Biochem, Littleton, CO) or ChIP-grade control rabbit IgG Sera (Abcam, Cambridge, U.K.). PCR primers were designed using web primer3 [61] and primer specificity checked against the human ref genome with primer-blast (NCBI) [62]. FOXP3 Chip Primer Sequences:

FP3_BR_F:GTTTTTCCTTCCAGCCTTTTTCCT. FP3_BR_R:TCCCCACGTAAATGTTTTGCTTTT. Control_F: TAATAGTGAGTCAGGAGCCAAACC. Control_R: CGGCAGAATGCTAAGGTATTTGTT. Zeb2 TSS (hg19 coordinates): chr2:145,277,958. FOXP3 binding region Zeb2: chr2:145,209,383-145,210,451. (68 kb downstream of the Zeb2 TSS and ∼31 kb downstream of the ChIP Control region). ChIP Control region: chr2:145,241,295-145,241,488. (36.567 downstream of the Zeb2 TSS). qPCR reactions were performed in triplicate using FastStart SYBR Green master mix (Roche Life sciences). The relative enrichment of target regions in FOXP3-and control IgG immunoprecipitated material relative to input chromatin was calculated using the 2^−ΔΔCT^ method.

### Plasmid construction

#### Promoter functional assay

The ZEB2 promoter from −1.7 kb to + 0.14 kb relative to the ZEB2 transcriptional start site (TSS) was cloned upstream of the Luciferase reporter gene in pGL4.10 (Promega Corp; Madison WI). A 1.6 kb fragment of intron2 of human ZEB2 containing the FOXP3 binding region identified by ChIP assays (+ 67 kb to + 68.6 kb relative to the Zeb2 TSS) was cloned downstream of the ZEB2-promoter-Luciferase construct. Primers used for ZEB2 Promoter and Intron 2 binding region reporter assay: GRCh 37/hg19:

Prom_F(chr2:145,277,818145,279,696):GGGGGAGAGAGTTAATTTATCCAGC.

Prom_R:TCTTTGTGGGGAGGGATAATTGAAG.

Int2_F(chr2:145,209,322–145,210, 795): CACCATGGATCCCTTTCTGACCAGCA AGCAGTT.

Int2_R:AGGGAAGTCGACTGGAAAGAAGTTGTTGTCTTTTTG.

The promoter region was cloned into the NheI/XhoI site, while the putative ZEB2 FOXP3-binding region in Intron 2 was cloned into the BamHI/SalI site downstream of the luciferase gene to reflect the chromosomal architecture of ZEB2.

#### 3′UTR analysis

The putative miR-155 sites (http://34.236.212.39/microrna/) are detailed in Figure 3. ZEB2 3′UTR was excised from pCIneo-hRL-ZEB2 (kind gift from GJG Goodall) and cloned into the NotI/XhoI sites of the Dual Luciferase reporter vector, PsiCHECK2™ (Promega). Primers used for generating ZEB2 3′UTR and truncations (NCBI: NM_014795).

3′UTR_F^*^(4325bp)CACCATCTCGAGCTAGTGGAGTTGGAGCTGGG.

3′UTR_R^**^(5582bp)CCTCACTAAAGGGAAGCGGCCGCTCTAG.

Δ1_F uses 3′UTR F Primer;

Δ1_R(5264):CTGGGAAGCGGCCGCGCCCAAATGATCAACGTCATG.

Δ2_F(4475):CACCATCTCGAGTACTTATGTATCACTACAAAC;

Δ2_R(5618)CTGGGAAGCGGCCGCTCATTAACTACATTCTTAGTTTG.

Δ3_F uses Δ2- Primer.

Δ3_R uses Δ1R primer.

ZEB2 sequences are in bold. ^*^ZEB2 3 ′UTR F primer incorporates: Xho1 restriction sequence from position 2033–2037 bp in vector pCIneo hRL-ZEB2 and ZEB2 3 ′UTR sequence starting at position 4325bp (bold) (NCBI: NM_014795). ^**^ ZEB2 3 ′UTR R primer incorporates: Not1 restriction sequence from position 3324 in pCIneo hRL-ZEB2, through to ZEB2 3 ′UTR (NCBI: NM_014795) position 5582bp. Construct Δ1 used the ZEB2 3′UTR forward primer reverse primer (Δ1_R) whose homology with ZEB2 3′UTR ends at 5264bp. This truncates the 3′UTR by 318 bp and deletes putative miR-155 sites 3 and 4 at positions 5285bp and 5530 bp respectively. The full-length ZEB 3′UTR used in these experiments was from position 4325–5582bp (NCBI: NM_014795).

#### miR-155 mutagenesis

Mutations of the putative miR-155 consensus sequences were carried out using the QuickChange™ mutagenesis method (Stratagene) using PCR primers (NCBI: NM_014795, mutation in lower case). We introduced a BsrG1 restriction site to ablate miR-155 site 1 and a Hind111 restriction site to ablate miR-155 site 2: Site 1 BsrG1- F (seq start 4325)

CACCATCTCGAGGGAGTTGGAGCTGGGTATTGTTAAAAACTtgtacaTGCAAAAATTTTGTACAG; Site 1 3′UTR- R (seq start 5618) CTGGGAAGCGGCCGCTCATTAACTACATTCTTAGTTTG; Site 2 HindIII- F (seq start 4416) CCTGTGTTTAATaagcttTATACTTTAAGC; Site 2 HindIII- R (seq start 4445) GCTTAAAGTATAaagcttATTAAACACAGG.

#### Lentiviral vectors

We have described construction of LV-411-GFP, LV-411-FOXP3 [26, 53] and LV-FOXP3-KD (LV-T-sh-) previously [53].

### Transfection, lentiviral packaging and transduction of cells

#### Transient transfection

BT549 or MDA-MB-231 cells were seeded in RPMI or HDMEM, 10% FCS (no antibiotics) media respectively, in 6-well plates for protein, and RNA quantitation and Invasion Assays and in quadruplicate in 96 well plates (Essen ImageLock™, Essen Bioscience, UK) plates for migration assays, three hours before transfection. MCF10A cells were seeded in medium as described above (Cell Culture section) in 24 well plates for miR-inhibitor experiments. Cells were transfected using Lipofectamine^®^ RNAiMAX reagent (Life Technologies) with either Control miR (Life Technologies mirVana^®^ miRNA Mimic Negative Control (#1) or hsa-miR-155-5p (Life Technologies mirVana^®^ miRNA mimic MC12601) at a final concentration of 20 nM. For silencing experiments, cells were transfected, using Lipofectamine^®^ RNAiMAX reagent (Life Technologies), with: Life technologies Silencer^®^ Select Pre-designed siRNAs to ZEB1 (: #1 ID: s229970, #2 ID: s229971, #3 ID: s229972) or ZEB2 (#1 ID: s19032, #2 ID: s19033 or #3 ID: s19034) or siRNA control (si-control) (ID: Silencer^®^ Select Negative control No.1 ID: 4390843) and for migration assays and HMEC transfections: Dharmacon (Dharmacon, GE Healthcare, Millenium Science Pty Ltd, Vic, Australia) siGenome siRNAs to huZEB1 (D-006564-05) or huZEB2 (D-006914-01) or si non targeting control (D-001210-03) at a final concentration of 25 nM. For miR-inhibitor experiments, MCF10A cells (8 × 10^4^) were seeded in 0.5 ml MCF10A culture medium and later the same day transfected using Lipofectamine^®^ RNAiMAX reagent (Life Technologies) with miR-155 inhibitor (Dharmacon miRIDIAN microRNA human hsa-miR-155-5p hairpin inhibitor cat# IH-300647-06, Millenium Science Pty Ltd, Vic, Australia) or control inhibitor (Dharmacon miRIDIAN microRNA hairpin inhibitor negative control #1 cat# IH-001005-01-05 Millenium Science Pty Ltd, Vic, Australia) at a final concentration of 10 nM. 72 hours after transfection, cells were transfected a 2nd time and incubated for a total time of 5 or 7 days, after which time total RNA was purifed (Qiagen miRNeasy Mini Kit). For ZEB2-3′UTR/miR analyses, HEK293T cells were plated in 96-well plates in triplicate and transfected using Lipofectamine^®^ 2000 reagent (Life Technologies) with PsiCHECK2™-ZEB2 3′UTR reporter (full-length, truncated or mutation constructs) or positive control PsiCHECK2™-SATB1 3′UTR reporter together with either Control-miR or hsa-miR-155-5p (Shanghai GenePharma) final concentration of 20 nM for 24 hr prior to Dual Luciferase Assay (Promega). For ZEB2 promoter analyses, BT549 cells stably expressing GFP or FOXP3 were transfected with pGL4.10 (Firefly luciferase reporter, Promega) constructs together with pGL4.74 (Renilla luciferase reporter, Promega) using Lipofectamine^®^ 2000 reagent (Life Technologies) as per the manufacturer’s protocol. For migration and invasion assays and ZEB2-3′UTR/miR analyses, cells were transfected overnight. For total RNA purification (Qiagen miRNeasy Mini Kit) or protein isolation cells were transfected and incubated at 37°C, 5% CO_2_ for 48 hrs or 72 hr respectively.

#### Lentiviral packaging and transduction

Lentiviral packaging as described previously [53]. BT549 transduction with LV411-GFP and LV411-FOXP3 (LV-GFP and LV-FOXP3 respectively) described previously [26]. HMECS seeded at a density of 5 × 10^4^ in 6 well plates were transduced with LVFOXP3 KD or LV Control lentivirus at an MOI of 1 in the presence of Polybrene (8 ug/ml Sigma, H9268). For immunofluorescence experiments, HMECS were seeded at a density of 2 × 10^4^ in glass-bottom 8-well chamber slides (Thermo Scientific™ Nunc™ Lab-Tek™ II Chamber Slide™ System, Thermo Fisher Scientific) and transduced with lentivirus. 24 hours after lentiviral transduction of HMECS, medium was removed and replaced with fresh medium. 72 hr after lentiviral transduction, RNA was isolated or for immunofluorescence experiments, cells were treated as described below. The percentage of GFP + lentiviral transduced HMECs was routinely assessed to be >80% but GFP + cells were not isolated.

### Migration and invasion assays

BT549 (2–3 × 10^4^) and MDA-MB-231 (1 × 10^4^) cells were seeded in 96 well plates 3 hours prior to transfection. 4 technical replicates were carried out for all transfections. For migration assays, 20 hours after transfection, scratch wounds were made (Essen Woundmaker™, Essen Biosciences) and immediately thereafter real-time cell migration was imaged over a 45–50 hour time period (IncuCyte™ ZOOM or IncuCyte™, Essen Biosciences). Relative Wound Density was calculated using the custom algorithms supplied with the IncuCyte™ software. These algorithms are capable of identifying the wound region and provide visual representations of the segmentation parameters. Cell type specific Processing Definition algorithms were used to analyse the data. From the data generated, Relative Wound Density for 4 technical replicates over the 45–50 hour period was plotted. Relative Wound Density data analysed at 40 hours (minus data from 0 hours) was collected from 4–6 experiments for BT549 cells) and from 3 experiments at 30 hours for MDA-MB-231 cells and the means generated from data were also plotted. For Invasion assays, low passage BT549 cells were transfected as described above. 16 hours after transfection, cells were serum starved for 24 hours (0.5% FCS). 96 well Invasion chambers (Cultrex^®^ Cell Invasion Assay, Trevigen Inc, MD, USA) were coated with 0.1x Basement Membrane Extract (Cultrex^®^ BME) according to the manufacturer’s instructions for four hours at 37°C 5% CO_2_ and 1 × 10^4^ cells were seeded per well, in triplicate, in low serum medium. 28 hours later, cells that had passed through the BME and adhered to the bottom assay chamber containing normal medium (10% FCS) were treated with Calcein-AM (Cultrex^®^ Cell Invasion Assay) according to manufacturer’ s instructions and fluorescence read at 485 (Excitation) 520 nm (Emission) using a Victor™ 2030 Multi Label Reader (Perkin Elmer). Calcein-AM is internalised by cells and is then cleaved internally to generate free Calcein which fluoresces brightly. The Relative Fluorescence Units (RFU) obtained were converted to cell number using a standard curve generated for each experiment and then used to quantitate the percentage of cells that have invaded calculated according to the Cultrex^®^ Cell Invasion Assay instructions and using cell free wells to normalise for background substrate fluorescence. Experiments were carried out four times.

### Luciferase assays

Dual Luciferase Assays were carried out according to the manufacturer’s instructions (Promega). Luminescence was measured on a Veritas Microplate Luminometer (Promega). Relative signals were calculated as ratios of reporter Renilla luciferase to intra-plasmid transfection normalisation reporter Firefly luciferase activities for PsiCHECK2™-ZEB2 3′UTR (full-length, deletion and mutation constructs) and PsiCHECK2™-SATB1 3′UTR analysis and as ratios of Firefly luciferase to Renilla luciferase activities for ZEB2 promoter analysis. All experiments were repeated at least three times.

### Quantitative RT-PCR

Total RNA from cell lines was isolated using Qiagen miRNeasy^®^ Mini kit following the manufacturer’s protocol. For mRNA quantitation, cDNA was generated from total RNA using the Qiagen QuantiTect kit. KAPA SYBR^®^ FAST qPCR kit was used for the subsequent quantitative real-time PCR. For micro RNA quantitation Taqman Assays (Taqman microRNA Assay hsa-miR-155: Cat# 4427975 Assay ID 002623, Taqman microRNA Assay hsa-miR-200b: Cat# 4427975 Assay ID 002251, Taqman microRNA Control Assay: RNU-24 Cat# 4427975 Assay ID 001001, Life Technologies) were used to generate miR-specific cDNAs and for subsequent quantitative real time PCR. All reactions for real -time PCR were run in triplicate using a Qiagen Rotor-Gene Q and the means of the threshold cycles (Cts) for the triplicate samples were determined and used for subsequent quantitation. A standard curve, using a template diluted (6 dilutions) over 3 orders of magnitude and plotted against the resulting the Ct values, to determine amplification efficiency, was generated for all of the mRNAs and microRNAs and for the reference mRNA RPL13a and microRNA reference RNU-24. The standard curve method for relative quantitation was then used to determine the relative abundance of each mRNA or miRNA normalised to its reference gene or reference miRNA respectively. RPL13a was chosen as the reference gene for mRNAs as its expression varied very little across the cell types and conditions used in these experiments. RNU-24 was used as the normaliser for miRNAs as its expression varied the least in optimisation studies across a range of cell types and conditions compared with other potential miRNA normalisers tested (miR-24, RNU6B, RNU44, data not shown). Data were analysed using the Q-Gene software as described in the manufacturer’s protocol. Experiments were repeated a minimum of three times. Primers used for KAPA SYBR^®^ FAST qPCR were designed using Primerbank (https://pga.mgh.harvard.edu/primerbank/) unless otherwise stated.

RPL13A F: (61) GCCATCGTGGCTAAACAGGTA.
RPL13A R: (195) GTTGGTGTTCATCCGCTTGC
ZEB2 F: (295) AACAACGAGATTCTACAAGCCTC.
ZEB2 R: (470)TCGCGTTCCTCCAGTTTTCTT
ZEB1 F: (567) TTACACCTTTGCATACAGAACCC.
ZEB1 R: (666) TTTACGATTACACCCAGACTGC
SATB1 F: (142) ACAGGTGCAAAAATGCAGGGA.
SATB1 R : (235) GCGTTTTCATAATGTTCCACCAC.
E-Cadherin F: (600) GTGGCCCGGATGTGAGAAG.
E-Cadherin R: (837) GGAGCCCTTGTCGGATGATG

FOXP3 qPCR Primers: FOXP3 primers were designed to span FOXP3 transcript variant 1 mRNA exons 4–6 (https://www.ncbi.nlm.nih.gov/nuccore/NM_014009.3, updated 2018) FOXP3 Exons 4–6 F: (402) CACCACCGCCACTGGGGTCT. FOXP3 Exons 4–6 R: (564) TCTGGGGCACAGCCGAAAGG

### Western blots

Cells were lysed in RIPA buffer (50 mM Tris/HCL (pH7.5) 150 mM NaCl, 1 mM EDTA,1% NP-40, 0.25% sodium deoxycholate, 0.1% SDS) containing Complete Protease Inhibitor cocktail (Roche) and Pefabloc ((AEBSF) Sigma Aldrich). Bio-Rad Protein Assay kit was used for protein quantitation. Protein (30 ug) was loaded onto 4%–15% Mini-PROTEAN^®^ TGX™ gels (Bio-Rad). Overnight transfer onto nitrocellulose membrane at 30 V in Towbin buffer (25 mM Tris base, 192 mM glycine) containing 10% methanol. ZEB2, (anti-SIP1 antibody, clone 6E5, Active Motif, Cat# 61095) Vimentin (anti-Vimentin antibody, R28, Cell Signalling, Cat# 3932) and E-Cadherin (anti E-Cadherin antibody, BD Transduction Laboratories, Cat# 610181) detection using PBST (PBS + 0.1% Tween20) containing 5% skim milk. For ZEB1 (anti-Zeb1 antibody E-20 Santa Cruz Biotechnology Cat# sc-10572) detection, membranes were blocked with 1X Roche Blocking Reagent for nucleic acid hybridization (Roche, Cat# 11096176001). Normalisation was carried out with either anti-αTubulin antibody (Rockland, Cat# 600-401-880) (used for ZEB1 and ZEB2 normalisation) or with anti-β-Actin antibody (Cell Signalling, Cat# 4967) (used for E-cadherin and Vimentin normalisation) for each of the membranes. Secondary antibodies were goat anti-mouse-, goat anti-rabbit- or mouse anti-goat-HRP (Rockland). Quantitation of bands was carried out using Image J software and abundance of protein relative to the appropriate normalisation protein was determined.

### Proliferation assays

BT549 (2.5 × 10^4^) cells were transfected (as described above) in a 96 well plate, using 4 technical repeats. 48 hours after transfection proliferation was measured by colourimentric analysis using the CellTiter 96^®^ Aqueous One solution Cell Proliferation Assay kit, according to manufacturer’s instructions (Promega). Absorbance was measured one hour after addition of the reagent at 490 nm in a Opsys MR 96-well plate reader (Dynex Technologies) and the average absorbance was subtracted from the no cell control to determine the corrected absorbance. The quantity of Formazan product measured by absorbance at 490nm is directly proportional to the number of living cells. Experiments were repeated three times.

### Immunofluorescence

HMEC cells (2 × 10^4^) or BT549 (4 × 10^4^) were cultured in glass-bottom 8-well chamber slides (Thermo Scientific™ Nunc™ Lab-Tek™ II Chamber Slide™ System, Thermo Fisher Scientific) coated with 5 ug/ml Human Recombinant Fibronectin ((R&D systems, Australia) in medium at 37° C/5% CO_2_ in a humidified incubator. Cells were fixed with 3.7% formaldehyde for 10 min room temperature, washed twice with PBS and permeabilised with 0.1% TritonX-100 for 10 min at room temperature. Permeabilised cells were incubated with blocking buffer (1% BSA/PBS) for 1h at room temperature, before either Alexa Fluor 647 conjugated – anti-E-cadherin (1:20, Cat# 560062, E-cadherin Alexa-Fluor^®^647 mouse anti-E-cadherin Clone 36, BD Pharmingen, BD Biosciences, San Jose CA) or anti-vimentin (1:50, Cat#9856, D21H3 XP Rabbit mAb AlexaFluor^®^ 647 Conjugate, Cell Signaling Technology^®^ Danvers MA) diluted in blocking buffer were applied and incubated overnight at 4° C. Samples were then washed twice with PBS, counter stained with Alexa Fluor^®^ 568Phalloidin (Cat#A12380, Cell SignalingTechnology^®^ Danvers, MA) in blocking buffer for 45 min, before being air dried briefly and mounted using ProLong^®^ Gold Antifade Fluorescent reagent (Thermo Fisher Scientific), containing 4′,6-diamidino-2-phenylindole (DAPI) for nuclear staining.

Confocal images were collected on a Leica TCS-SP5 upright confocal microscope using a 40 × oil-immersed objective with 2.5 × optical zoom applied using identical exposure settings for each antibody fluorophore for FOXP3 KD and Control lentivirus transduced cells. Images are compositions of 12 optical sections of a Z-stack, recorded at 250 nm per vertical step with four times line averaging. ADOBE PHOTOSHOP (Adobe Systems) and FIJI (NIH) was used for processing of images.

### Statistical analysis

For all experiments, unless otherwise stated, statistical significance between two groups was determined using a Student’s *t* test performed using GraphPad Prism 6. A *p* value < 0.05 indicates a statistically significant difference: ^*^P < 0.05, ^**^P < 0.01, ^***^P < 0.001.

## SUPPLEMENTARY MATERIALS FIGURES


