# Supplementary material for: FOXP3 and miR-155 cooperate to control the invasive potential of human breast cancer cells by down regulating ZEB2 independently of ZEB1
Source: Oncotarget. 2018 Jun 12;9(45):27708–27. doi: 10.18632/oncotarget.25523 (PMC6021232; doi:10.18632/oncotarget.25523)
Supplement: Supplementary file 1 [file oncotarget-09-27708-s001.pdf]

FOXP3 and miR-155 cooperate to control the invasive potential of human breast cancer cells by down regulating ZEB2 independently of ZEB1

SUPPLEMENTARY MATERIALS

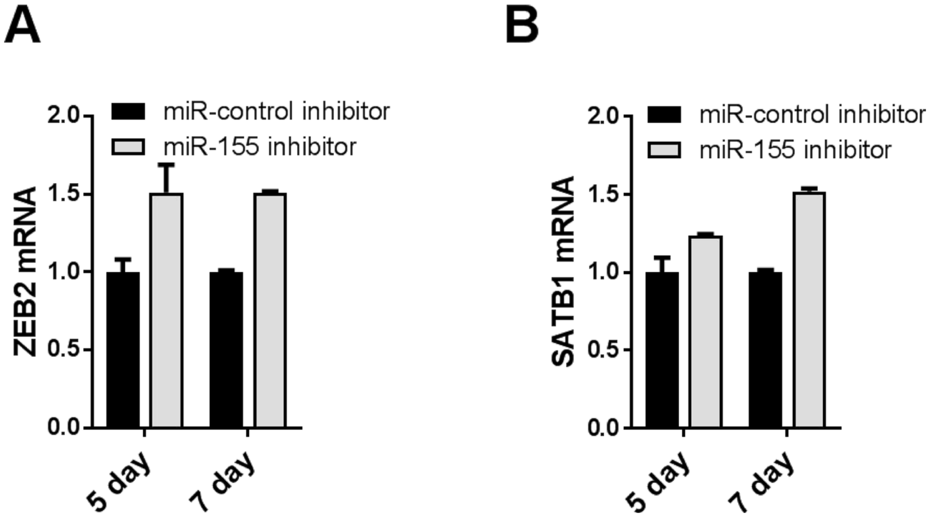

Supplementary Figure 1: De-repression of endogenous ZEB2 and SATB1 by inhibition of miR-155 targeting. (**A**) Relative abundance of ZEB2 mRNA 5 days and 7 days after transfection of MCF-10A cells with miR-155 inhibitor or miR-Control inhibitor. Relative abundance of ZEB2 mRNA normalised to reference gene RPL13A is plotted. Quantitative real -time PCR reactions were in triplicate and the means of the threshold cycles (Cts) were used for quantitation. A standard curve to determine amplification efficiency was generated (see Methods section) for ZEB2 and for the reference gene RPL13a mRNAs. The standard curve method for relative quantitation was used to determine the relative abundance of ZEB2 mRNA normalised to the RPL13a reference gene. One experiment shown. (**B**) Relative abundance of SATB1 mRNA 5 days and 7 days after transfection of MCF-10A cells with miR-155 inhibitor or miR-Control inhibitor. Relative abundance of SATB1 mRNA normalised to reference gene RPL13A is plotted. Reactions for quantitative real -time PCR were run in triplicate, standard curves to determine amplification efficiency were determined for SATB1 and RPL13A reference and the standard curve method for determination of relative abundance of mRNA was as in (A) above. One experiment shown.

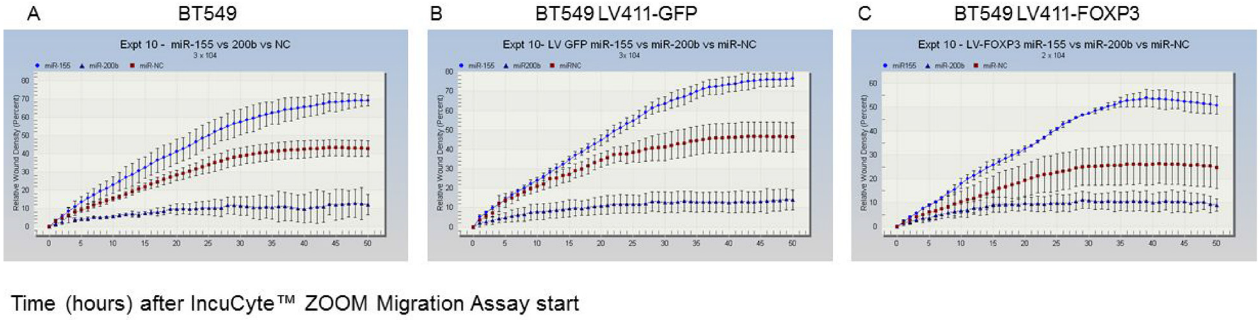

Supplementary Figure 2: Loss of ZEB2 leads to altered migration in human breast cancer cells. (**A**) Real-time cell migration in WT BT549 cells transfected with miR-155, miR-200b or miR-control (NC). Relative Wound Density (%) was calculated using the custom algorithms supplied with the IncuCyte™ software. Cell type specific Processing Definition algorithms were used to analyse the data (see Methods). Relative Wound Density data was generated for 4 technical replicates (± SEM) over the 50 hour period. ● miR-155, ▲ miR-200b, ■ NC. (**B**) Real-time cell migration in LV-GFP (LV411-GFP) BT549 cells transfected with miR-155, miR-200b or miR-control (NC). Relative Wound Density (%) was calculated using the custom algorithms as in (A) above. Relative Wound Density data was generated for 4 technical replicates (± SEM) over the 50 hour period. ● miR-155, ▲ miR-200b, ■ NC. (**C**) Real-time cell migration in LV-FOXP3 (LV411-FOXP3) BT549 cells transfected with miR-155, miR-200b or miR-control (NC). Relative Wound Density (%) was calculated using the custom algorithms as in (A) above. Relative Wound Density data was generated for 4 technical replicates (± SEM) over the 50 hour period. ● miR-155, ▲ miR-200b, ■ NC.
